# Increased Ventromedial Prefrontal Cortex Activity in Adolescence Benefits Prosocial Reinforcement Learning

**DOI:** 10.1016/j.dcn.2021.101018

**Published:** 2021-10-02

**Authors:** Bianca Westhoff, Neeltje E. Blankenstein, Elisabeth Schreuders, Eveline A. Crone, Anna C.K. van Duijvenvoorde

**Affiliations:** a Institute of Psychology, Leiden University, Wassenaarseweg 52, 2333 AK Leiden, Netherlands; b Leiden Institute for Brain and Cognition, Leiden, Netherlands; c Amsterdam UMC, Vrije Universiteit Amsterdam, Child and Adolescent Psychiatry, de Boelelaan 1117, Amsterdam, Netherlands; d Department of Clinical, Neuro and Developmental Psychology, Vrije Universiteit, Amsterdam, Netherlands; e Erasmus School of Social and Behavioural Sciences, Erasmus University Rotterdam, Netherlands

**Keywords:** Adolescence, Prosocial, Reinforcement learning, Prediction error, Ventromedial prefrontal cortex, Cognitive empathy

## Abstract

Learning which of our behaviors benefit others contributes to forming social relationships. An important period for the development of (pro)social behavior is adolescence, which is characterized by transitions in social connections. It is, however, unknown how learning to benefit others develops across adolescence and what the underlying cognitive and neural mechanisms are. In this functional neuroimaging study, we assessed learning for self and others (i.e., prosocial learning) and the concurring neural tracking of prediction errors across adolescence (ages 9–21, N = 74). Participants performed a two-choice probabilistic reinforcement learning task in which outcomes resulted in monetary consequences for themselves, an unknown other, or no one. Participants from all ages were able to learn for themselves and others, but learning for others showed a more protracted developmental trajectory. Prediction errors for self were observed in the ventral striatum and showed no age-related differences. However, prediction error coding for others showed an age-related increase in the ventromedial prefrontal cortex. These results reveal insights into the computational mechanisms of learning for others across adolescence, and highlight that learning for self and others show different age-related patterns.

## 1 Introduction

Adolescence is a developmental phase that is characterized by transitions in social connections, and moreover, a phase during which social cognitive skills are acquired and/or improved (Blakemore and Mills, 2014, Casey et al., 2008, Crone and Dahl, 2012, Sawyer et al., 2018). As social acceptance and approval from peers often result from displaying prosocial behaviors, for adolescents establishing their social network it is key that they learn to help or benefit others (Steinberg and Morris, 2001). That is, to be able to behave in a prosocial manner, individuals need to learn which actions would result in positive outcomes for others. This type of learning is also referred to as prosocial learning (Lockwood et al., 2016, Sul et al., 2015). Generally speaking, learning from actions and outcomes is an important part of cognitive development and continues to improve in adolescence (Bolenz et al., 2017, Nussenbaum and Hartley, 2019, Peters et al., 2014a, Peters et al., 2016). For adolescents, an especially salient environment that requires learning about the consequences of their actions is the interpersonal context (Blakemore and Mills, 2014, Nelson et al., 2005, Sawyer et al., 2018). Therefore, it is expected that especially prosocial learning shows improvements in adolescence. The goal of the current study was to unravel age-related differences in learning to benefit others using a prosocial learning context across adolescence.

The vast majority of recent neuroscientific studies investigating learning make use of formal reinforcement learning (RL) models. These models calculate individuals’ prediction errors (PEs) – the difference between expected and actual outcomes - over the course of learning. These PEs drive learning via a learning rate, which quantifies to what extent these PEs affect subsequent actions. Consequently, RL models and the resulting PEs enable studies to examine the neural tracking of value-guided decision-making. Neuroscientific studies demonstrated that PE coding in a probabilistic reinforcement task context is associated with activation in the ventral striatum, as well as the medial prefrontal cortex (mPFC) (see for reviews e.g., Cheong et al., 2017, Joiner et al., 2017, Lockwood and Klein-Flügge, 2020, Olsson et al., 2020, Ruff and Fehr, 2014 and Cheong et al., 2017, Joiner et al., 2017, Lockwood and Klein-Flügge, 2020, Olsson et al., 2020, Ruff and Fehr, 2014 and Cheong et al., 2017, Joiner et al., 2017, Lockwood and Klein-Flügge, 2020, Olsson et al., 2020, Ruff and Fehr, 2014). Developmental studies using RL models found that adolescents show similar neural tracking of PEs as adults when learning stimulus-outcome associations. However, the developmental patterns are inconsistent: some studies have reported elevated or lowered PE activity in the ventral striatum and connected structures in mid-adolescents relative to children and adults (Cohen et al., 2010, Davidow et al., 2016, Hauser et al., 2015, Jones et al., 2014), but this is not replicated in all studies (Christakou et al., 2013, van den Bos et al., 2012). Furthermore, age-related differences have been found in functional connectivity between the ventral striatum and mPFC, here referred to as ventromedial PFC (vmPFC), in relation to learning (van den Bos et al., 2012), suggesting that age-related improvements in learning are associated with stronger neural coupling between subcortical and cortical brain regions (van Duijvenvoorde et al., 2016, van Duijvenvoorde et al., 2019). Taken together, previous studies point to the ventral striatum and medial prefrontal cortex as important brain areas for learning in non-social environments.

Previous studies investigating the neurocomputational mechanisms of prosocial learning have investigated whether the same neural signaling occurs for PEs for others as for self. Recently, in adults, it was found that PE tracking for both learning for others as for self occurred in the ventral striatum (Lockwood et al., 2016). However, the subgenual anterior cingulate cortex (sgACC) specifically coded PE tracking for learning for others, and these prosocial learning signals were predicted by cognitive empathy. That is, more empathic people showed more activity in the sgACC when learning to benefit others. Cognitive empathy – the ability to understand the emotional states of others (Netten et al., 2015, Pouw et al., 2013) - shows pronounced changes in adolescent development and relates positively to prosocial behaviors such as trust and reciprocity (Dumontheil et al., 2010, Eisenberg et al., 1995, van de Groep et al., 2018). Therefore, we aimed to extend prior work by Lockwood et al. (2016) by investigating the neural tracking of PEs for others, and its relation with individual differences in cognitive empathy, in an adolescents sample with participants aged between 9 and 21 years.

In the current study, we adopted a prosocial learning task (Lockwood et al., 2016) in which participants could learn to obtain rewards for themselves, others, or no one. We administered this task to 74 adolescents between ages 9–21 years to examine age-related differences in learning for self and others, combined with functional neuroimaging (fMRI) for neural tracking of PEs. We use the term adolescence for this broad age range, based on definitions that mark adolescence from the onset of puberty to the age when one reaches independence from parents (i.e., approximately 9–24 years; e.g., Sawyer et al., 2018). Based on prior studies, we performed regions-of-interest analyses for the ventral striatum, sgACC, and vmPFC. We expected that adolescents, similar to adults, would show PE related neural activity when learning both for self and others in the ventral striatum (Lockwood et al., 2016), and in the sgACC and possibly vmPFC when learning for others more than when learning for self (Christopoulos and King-Casas, 2015, Lockwood et al., 2016). For learning for self, research has remained inconclusive whether this activity peaks in mid-adolescence (Cohen et al., 2010, Davidow et al., 2016) or shows no age-related differences (van den Bos et al., 2012). Therefore we explored linear as well as non-linear (quadratic) age effects. We predicted that sgACC and vmPFC activity for prosocial learning would increase with age, based on prior studies showing age-related improvements in social-cognitive perspective-taking (Dumontheil et al., 2010). Finally, consistent with (Lockwood et al., 2016), we expected that individual differences in cognitive empathy would relate to neural tracking of PEs for others.

## 2 Methods and materials

### 2.1 Participants

A total of 76 participants between ages 9 and 21 took part in this study. Participants were recruited through schools and local advertisements, as well as from participation in a previous study. Two participants were excluded from analyses because they were either diagnosed with a psychiatric disorder at the time of testing (*n* = 1) or because the session was stopped early due to discomfort in the scanner (*n* = 1). We did not exclude participants based on task performance; there were no significant outliers in task performance (i.e., > 3 SD) in any of the conditions. Four participants missed one run of the task, due to technical issues (*n* = 2), or discomfort in the scanner (*n* = 2). These four participants were maintained with the available data in all analyses. The final sample included 74 healthy participants (39 female, *M*_*age*_ = 15.64, *SD*_*age*_ = 4.18, range = 9.03–21.77 years, see Fig. S1 for an overview of the number of participants across ages). The IQ scores, estimated with the Similarities and Block Design subtests of the WISC-III and WAIS-III, fell within the normal range (*M*_IQ_ = 110.24, *SD*_IQ_ = 10.37, range = 87.50–135.00), and did not correlate with age (*r*(72) = − 0.11, *p* = .353).

The local institutional review board approved this study (reference: NL56438.058.16). Adult participants and parents of minors provided written informed consent, and minors provided written assent. All anatomical scans were cleared by a radiologist and no abnormalities were reported. Participants were screened for MRI contraindications and psychiatric or neurological disorders, and had normal or corrected-to-normal vision.

### 2.2 Prosocial learning task

**Fig. 1:**
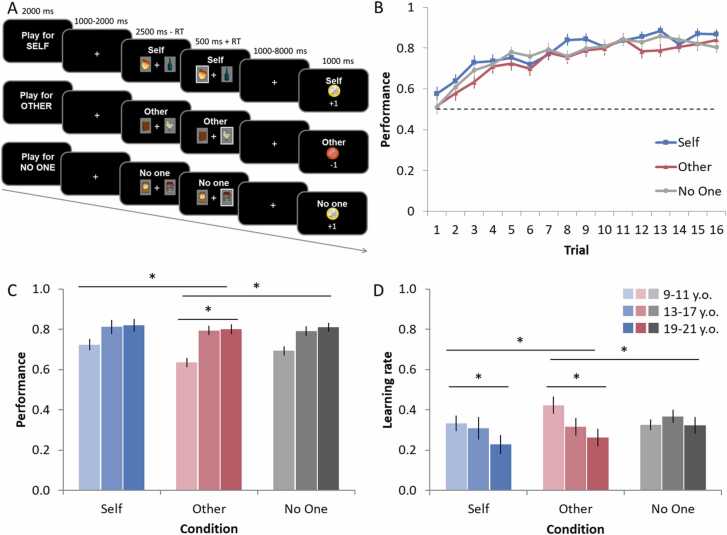
**Prosocial learning task and behavioral data**. **(A)** Participants played a two-choice probabilistic reinforcement learning task in which outcomes resulted in monetary consequences for themselves (Self condition), for an unknown other participant in the experiment who could not reciprocate (Other condition), or for No One. **(B)** Group-level performance across trials (learning curves) per condition, averaged across blocks. Performance represents the fraction of selecting the stimulus with a high reward contingency. The dashed line indicates performance at chance level (0.5). **(C)** Performance per condition per age cohort, averaged across the entire task. In all conditions, performance improved across trials, but an age-related increase was only observed when learning for others. Note that age is used as a continuous variable in all analyses but is visualized as age cohorts for illustrative purposes. The age-related increase was greater for the Other than for the Self and No One condition. **(D)** Learning rates per condition per age cohort. Age-related decreases in learning rates are only observed in the Self and Other condition. The age-related decrease in learning rate was greater in the Other compared to the Self and No One condition. Asterisks indicate significant effects. Error bars represent standard error of the mean (s.e.m.).

Participants played a two-choice probabilistic reinforcement learning task (prosocial learning task) in the MRI scanner (see Fig. 1A). Participants were instructed to make a series of decisions between two pictures. One picture was associated with a high probability of winning 1 point, the other picture with a high probability of losing 1 point. The exact probabilities were 75% and 25% but were unknown to the participant. After the decision, participants were presented with the outcome to enable them to learn the reward contingencies.

The participants played the task in three different conditions: for themselves (Self), for an unknown other participant (Other), or for No One. The latter condition was added as a control condition based on Lockwood et al. (2016). Participants did not meet the other person, but were told that the other person was a peer also participating in the experiment who i) would not play the same game for them, ii) did not know who played for them (see Participant instructions in the Supplementary Information). Each block started with an instruction screen that indicated who would receive the outcomes (Self, Other, or No One) for 2000 ms. This was followed by the presentation of two stimuli for 2500 ms during which participants were required to select one of these. The stimuli were common objects, such as chairs, apples, and shoes (see also (van den Bos, 2009)). If no response was given within the time frame, the text “Too late” appeared in the middle of the screen, and these trials were excluded from analyses.

A selection frame around the chosen picture confirmed the response and remained visible for the duration of the interval and an additional 500 ms. A fixation screen (duration randomly jittered between 1000 and 2000 ms) preceded the outcome of their choice (+1 point or −1 point; 1000 ms). A randomly jittered fixation screen (1000–8000 ms) was shown after the outcome before the two pictures were presented again. The screen position of the stimulus (left or right) was counterbalanced across trials. Participants were instructed that the position of the stimulus did not matter, to encourage them to learn the reward contingencies regardless of stimulus position.

There were 144 trials in total, 48 for Self, 48 for Other, and 48 for No One, presented in three blocks of 16 trials. Each block began with a new pair of pictures. Participants completed three separate fMRI runs with a short break in between, each with one block of 16 trials per condition. The order of the conditions was counterbalanced across runs and between participants.

Participants were instructed that the total number of points in the Self condition was converted to money (each point valued €0.25), which they would get paid out on top of their flat participation rate (€20 for 9–11 y.o., €25 for 13–17 y.o., and €30 for 19–21 y.o.). The minimum of this extra amount of money was €1 to avoid null scores, and the maximum was €12. Additionally, participants were instructed that their choices in the Other condition were paid out to a participant entering the experiment after them. Consequently, participants received an additional fee from a participant before them in the experiment (minimum €1 and maximum €12), but only at the end of the experiment. Finally, it was instructed that choices in the No One condition had no financial consequences.

### 2.3 Cognitive empathy

To assess cognitive empathy, participants completed the Interpersonal Reactivity Index (IRI; (Davis, 1983)). This widely used self-report questionnaire consists of 4 subscales (Perspective-Taking and Fantasy as cognitive empathy subscales; and Personal Distress and Empathic Concern as affective empathy subscales) with 6 items each. To create a measure of cognitive empathy, two subscales were combined (Pulos et al., 2004): the Perspective-Taking subscale (e.g., “I sometimes try to understand my friends better by imagining how things look from their perspective”, Cronbach’s alpha = 0.710) and the Fantasy subscale (e.g., “I really get involved with the feelings of the characters in a novel”, Cronbach’s alpha = 0.786). All items can be answered on a five-point Likert scale ranging from (0) not true at all to (4) completely true, and higher scores indicate higher levels of empathy. Cognitive empathy scores increased across age (*r* = 0.309, *p* = .008, see Fig. S8). One person did not fill in this questionnaire. This person was excluded from further analyses concerning measures of (cognitive) empathy. We used a Dutch adolescent version for all ages in our study, with items adapted for the youngest ages in the study (Hawk et al., 2013).

### 2.4 Procedure

Participants were accustomed to the MRI environment using a mock scanner, and received instructions on the prosocial learning task in a quiet laboratory room. Instructions for the task were displayed on a screen and read out loud by an experimenter. Participants completed 8 practice trials in each condition. In the scanner, participants responded with their right hand using a button box. Head movements were restricted with foam padding. The fMRI scan was accompanied by a high-definition structural scan. Questionnaires were filled out at their home prior to the scanning session, via Qualtrics (www.qualtrics.com).

### 2.5 Computational modeling of behavioral data

#### 2.5.1 Model fitting

We used MATLAB 2015b (The MathWorks Inc) for all model fitting and comparison. We modeled learning behavior in the Self, Other, and No One conditions separately, using a standard Rescorla-Wagner reinforcement learning (RL) model (similar to Lockwood et al., 2016) to obtain PEs and learning rates, which were subsequently used in behavioral and fMRI analyses. Simple RL models state that the expected value of a future action (Q_t+1_(i)) should be a function of current expectations (Q_t_(i)) and the difference between the actual reward that has been experienced on this trial (R_t_). The learning rate α, bounded between 0 and 1, determines how much the value of the chosen stimulus is updated based on the new outcome. In particular, the learning rate parameter speeds up or slows down the acquisition and updating of associations. Optimal learning rates differ between contexts and reinforcement structures (Nussenbaum and Hartley, 2019).

$$Q_{t+1}(i)=Q_{t}(i)+\alpha *\underset{prediction\;error}{\underset{︸}{\left[R_{t}-Q_{t}(i)\right]}}$$

To select an action based on the computed values, we used a standard softmax choice function. For a given set of parameters, this equation allows us to compute the probability of the next choice being “i”:

$$P_{t}(i)=\frac{e({\beta *Q}_{i,t})}{\sum_{j}e({\beta *Q}_{j,t})}$$

Beta (β) determines *how strongly* action probabilities are guided by their expected values (Q). Here, with larger β, actions are more deterministic and driven by expected values, resulting in selecting the option with the highest value. With lower β, actions are more random or exploratory. This parameter thus affects errors, where a decrease will lead to more random (i.e., less driven by expected values) choices. β did not differ between conditions, although with age, people were more strongly driven by expected values (see Fig. S2 for the β across age cohorts for each condition).

We used the maximum a posteriori (MAP) approach (Daw, 2011) for fitting the RL model to participants’ choices per condition. To facilitate stable estimation across subjects, we used weakly informative priors to regularize the estimated priors toward realistic ones. These weakly informative priors and estimation procedures were based on previous research (den Ouden et al., 2013), and included a Beta (1.2, 1.2) distribution for the estimated α (learning rate) parameter (0 < α < 1) and a Gaussian distribution (0, 10) for the estimated β parameter (− ∞ ≤ β ≤ ∞). Mean and confidence intervals for each of the fitted parameters across all subjects are displayed in Supplementary Table S1.

#### 2.5.2 Model comparison

Based on previous developmental findings (e.g., van den Bos et al., 2012) we compared an alternative model with two learning parameters (i.e., separate learning rates for gains and losses) in order to benchmark the performance of the one-learning parameter model (i.e., one learning rate). Model comparisons revealed that the one-learning parameter model had a superior fit to the behavioral data for each condition, according to the Bayesian Information Criterion (BIC) (see Fig. S3). This was the case in each condition for the majority of the participants (81.1% Self, 74.3% Other, 76.7% No One), in all age cohorts, see Fig. S3. In none of the conditions, the BIC difference scores (Fig. S3) were correlated with age (*r*_*s*_, all *p* values > 0.14).

#### 2.5.3 Simulations and parameter recovery

To assess whether computational model parameters could be successfully recovered, we simulated choice behavior for the range of learning rates and beta’s that we encountered in our dataset. That is, we simulated a new participant dataset based on the α and β values from our participants as input parameters. This resulted in a simulated dataset with 74 participants. Parameter recovery, as indicated with correlations between simulated and recovered learning rates and beta values per conditions, is presented in Fig. S4.

### 2.6 Behavioral analyses

To assess learning for Self, Other, and No One, and their developmental patterns in the prosocial learning task, we fitted logistic generalized linear mixed models (GLMMs) to decisions (correct coded as 1, incorrect as 0) for each condition separately. These analyses were conducted in R version 4.0.1 (R Core Team. 2020), using the lme4 package (Bates et al., 2014). Our GLMMs included fixed effects of Age in years (linear and quadratic), Condition, Trial, and all interactions. Since no significant main or interaction effects of age-quadratic were observed in the choice data, this term was dropped in the final presented behavioral models for model parsimony. In all models, participant ID entered the regression as a random effect to handle the repeated nature of the data. Where applicable, Trial was additionally included as a random slope per subject. We performed post hoc tests using the *emmeans* package (Lenth et al., 2021), as well as tests per condition to delineate Age x Trial x Condition effects.

Next we examined the estimated learning rates per condition. These parameters indicate how people updated the value of stimuli based on outcomes for Self, Others, and No One. Since learning rates were not normally distributed, we used a robust linear mixed effects model (RLMM, rlmer function, robustlmm package (Koller, 2016)) in R (see also Cutler et al., 2021), with Condition and Age linear as fixed main effects and interaction effects. We performed post-hoc tests per condition and pair-wise contrasts per Condition. In all GLMM and RLMM models, continuous independent variables were mean-centered and scaled, and categorical predictor variables were specified by a sum-to-zero contrast (e.g., sex: − 1 = boy, 1 = girl). P-values for the GLMM were generated by using the Anova log-likelihood ratio tables from the afex package (Singmann et al., 2020). For the RLMM models, the Satterthwaite-approximated degrees of freedom generated by the lme4 model in combination with the output of the RLMM, was used to generate P-values.

Finally, we assessed whether cognitive empathy related to learning performance, learning rate, and PE activation when learning for others. We ran (partial) spearman correlational analyses with learning for self and cognitive empathy as predictors using the package ‘ppcor’ (Kim, 2015).

### 2.7 fMRI acquisition

For acquiring (functional) MRI data, we used a 3T Philips scanner (Philips Achieva TX) with a standard eight-channel whole-head coil. The learning task was projected on a screen that was viewed through a mirror on the head coil. Functional scans were acquired during three runs of 200 dynamics each, using T2* echo-planar imaging (EPI). The volumes covered the entire brain (repetition time (TR) = 2.2 s; echo time (TE) = 30 ms; sequential acquisition, 38 slices; voxel size 2.75 × 2.75 × 2.75 mm; field of view (FOV) = 220 (ap) x 220 (rl) x 114.68 (fh) mm). The first two volumes were discarded to allow for equilibration of T1 saturation effects. After the learning task, a high-resolution 3D T1 scan for anatomical reference was obtained (TR = 9.76 msec, TE = 4.95 msec, 140 slices, voxel size = 0.875 × 0.875 × 0.875 mm, FOV = 224 (ap) x 177 (rl) x 168 (fh) mm).

#### 2.7.1 Preprocessing

Data were analyzed using SPM8 (Wellcome Department of Cognitive Neurology, London). Images were corrected for slice timing acquisition and rigid body motion. We spatially normalized functional volumes to T1 templates. Occasional framewise displacement > 3 mm occurred for 3 participants in 1–2 volumes. For those participants with frame-frame head motion > 3 mm, an extra regressor was included corresponding to each volume (*n* = 3, for maximum 2 volumes). All other participants did not exceed translational head movement more than 3 mm in any of the scans (*Mean* = 0.65 mm, *SD* = 0.059 mm). The normalization algorithm used a 12 parameter affine transform with a nonlinear transformation involving cosine basis function, and resampled the volumes to 3 mm^3^ voxels. Templates were based on MNI305 stereotaxic space. The functional volumes were spatially smoothed using a 6 mm full width at half maximum (FWHM) isotropic Gaussian kernel.

#### 2.7.2 General linear model

We used the general linear model (GLM) in SPM8 to perform statistical analyses on individual subjects’ fMRI data. The fMRI time series were modeled as a series of two events: the decision phase (Expected Value, EV) and the outcome phase (PE), convolved with a canonical hemodynamic response function (HRF). The onset of the choice (EV), and the onset of the outcome (PE) were both modeled with zero duration. Each of these regressors was associated with a parametric modulator taken from the computational model. At the time a stimulus was selected (decision phase) this was the chosen expected value, and at the time of the outcome, the PE. The PEs were estimated using each subject’s own alpha and beta from each condition. Trials on which participants did not respond were modeled separately as a regressor of no interest. Six motion parameters, and -if applicable- motion censoring regressors were included as nuisance regressors. We used the MarsBaR toolbox (http://marsbar.sourceforge.net) to visualize the patterns of activation, in clusters identified in the whole-brain results. Coordinates of local maxima are reported in MNI space. Our main hypotheses centered on PE coding. For completeness, effects of EV at choice onset are included in Supplemental Table S3. In addition, uncorrected T-maps of EV and PE effects are uploaded on Neurovault (https://neurovault.org/collections/EOTSVZYT/). For condition effects, we examined contrasts of Self versus Other in concordance with (Lockwood et al.(2016). Contrasts were obtained from a flexible factorial design with three levels (Self PE, Other PE, No One PE). Effects and conclusion remained the same when testing Self PE > Other PE + No One PE, and Other PE > Self PE + No One PE. In Supplemental Table S4 we include all contrasts between conditions within our ROIs. Whole-brain effects for main effects and between conditions are included in Supplemental Tables S2 and S5, respectively. Age effects (linear and quadratic) were tested in follow-up regressions.

#### 2.7.3 ROI selection and fMRI analyses

The a priori regions of interest (ROI) in which we test our main hypotheses were defined anatomically and based on previous research on (prosocial) learning and feedback processing (Lockwood et al., 2016, van den Bos et al., 2012, van Duijvenvoorde et al., 2014). In concordance with previous studies, masks were taken from an appropriate atlas. That is, the bilateral ventral striatum and vmPFC were determined by an anatomical mask from the Harvard-Oxford Atlas (van Duijvenvoorde et al., 2014, van den Bos et al., 2012, Braams et al., 2015, Peters and Crone, 2017), and the sgACC was defined as Brodmann areas (BA) 25 and s24 (Lockwood et al., 2016). The sgACC region and the ventral striatum are anatomically adjacent and partly overlapping (see Fig. S5), but significant peak activations in either ROI were not observed in these overlapping voxels. Coordinates for local maxima are reported in MNI space. Effects in our ROIs are reported at *p* < .05 FWE-small volume corrected (SVC). Predictions were tested while correcting for multiple comparisons (3 ROIs) by limiting the false discovery rate (FDR; (Benjamini and Hochberg, 1995)); all reported tests survived this correction. Explorative whole-brain analyses are reported in Supplemental Tables S2 and S5, and Fig. S6.

## 3 Results

### 3.1 Developmental differences in learning to obtain rewards for Self, Others, or No One

Results showed that, at the group level, participants were able to learn for Self, Other, and No One, as they performed above chance level in all conditions (0.5; *t* values > 13.0, all *p*s < 0.001, *df* = 73; Fig. 1B). Using a generalized linear mixed model (GLMM) on participants’ choice behavior over trials, we assessed age-related differences in performance when learning for Self, Other, and No One. Performance in the learning task improved linearly with age (main effect of Age linear, *p* = .001). Moreover, we observed that age-related differences in learning performance differed per condition (Age x Condition interaction, *p* = .005). Post-hoc analyses revealed that the age-related improvement in performance was larger when learning for Other than when learning for Self (*p* = .009) and when learning for No One (*p* = .02). The age-related improvements were similar for learning for Self and No One (*p* = .92). Similarly, we also observed age-related differences in learning curves across trials, which differed per condition (Age x Condition x Trial interaction, *p* = .007). Specifically, younger children learned more slowly (i.e., flatter learning curves) across trials when learning for others, but this age effect on trial was not observed for the Self and No One condition (Age linear x Trial, for Other condition, *p* < .001; Self and No One conditions: *p*s > 0.2; see Figs. 1C and S7). Together, these findings suggest that across adolescence prosocial learning shows a more protracted improvement than when learning for Self or No One.

Next, we examined participants’ learning rates to assess how they updated the value of stimuli on the basis of outcomes for Self, Others, and No One. That is, higher learning rates indicate that people adjusted behavior quickly towards recent feedback, whereas lower learning rates indicate a slower pace in updating in which outcomes across multiple trials are integrated. Using a robust linear mixed effects model, we assessed effects of Condition and Age (linear) in learning rates (Fig. 1D). We observed that learning rates for Self were lower than learning rates for Other ([Self vs Other], *b* = 0.02, *p* < .001) and for No One ([Self vs No One], *b* = − 0.03, *p* < .001). Learning rates for Other and for No One did not differ ([Other vs No One], *p* = .911). Moreover, we observed that learning rates decreased linearly with age (main effect of Age linear, *b* = − 0.04, *p* = .023), an effect that also differed across conditions. Specifically, learning rates decreased across age in the Other and Self condition, but more strongly across age for Other than for Self ([Other-Self]*Age, *b* = − 0.02, *p* < .001) and for Other than for No one ([Other-No One]*Age, *b* = 0.004, *p* = .004). Learning rates also decreased more strongly across age for Self than for No One ([Self-No One *Age, *b* = .019, *p* < .001). Learning rates did not differ across age in the No One condition (*p* = .08). Together, these findings show that for both learning for Self and Others, younger participants responded more to recent feedback, whereas older participants integrated feedback more over trials. Moreover, this age-related change was most pronounced in the Other compared to the Self and No One condition.

### 3.2 Identifying common and distinct coding of prediction errors for Self and Others

**Fig. 2:**
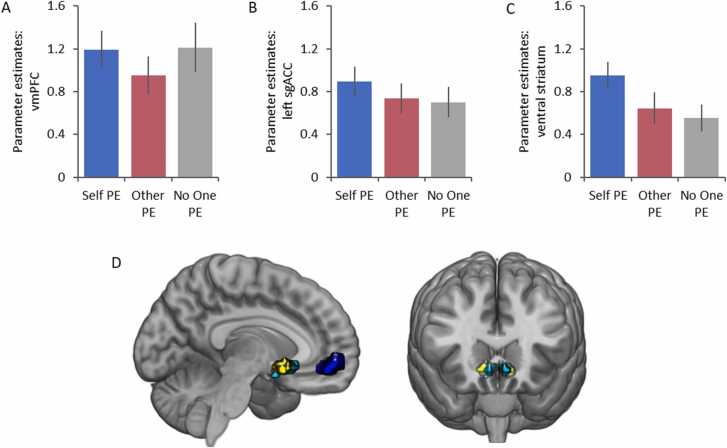
**Common prediction error (PE) coding in three regions of interest**. Shown are the responses to prediction errors for Self, Other, and No One in **(A)** the vmPFC, **(B)** left sgACC, and **(C)** ventral striatum. **(D)** Significant clusters of activation in the vmPFC (blue), sgACC (cyan), and ventral striatum (yellow). All images displayed at *p* < .05 FWE-SVC. (For interpretation of the references to color in this figure legend, the reader is referred to the web version of this article.)

To formally investigate the brain regions that were responding to PEs for Self, Others, and No One, we conducted a conjunction analysis to explore whether there were regions that commonly code PEs across all conditions. Common activation for PEs regardless of the beneficiary was observed in the vmPFC (MNI coordinates [*x* = − 9, *y* = 44, *z* = − 11], *Z* = 5.33, *k* = 136, *p* < .001, SVC-FWE), ventral striatum ([*x* = − 9, *y* = 11, *z* = − 11], *Z* = 5.05, *k* = 23, *p* < .001, SVC-FWE, and [*x* = 12, *y* = 14, *z* = − 8], *Z* = 4.43, *k* = 18, *p* < .001, SVC-FWE), and sgACC ([*x* = − 6, *y* = 14, *z* = − 8], *Z* = 5.47, *k* = 32, *p* < .001; and Self [*x* = 6, *y* = 17, *z* = − 8], *Z* = 4.60, *k* = 21, *p* *=* *.*001; and [*x* = 9, *y* = 8, *z* = − 14], *Z* = 3.67, *k* = 2, *p* *=* *.*029, SVC-FWE) (see Fig. 2). These findings show that all regions of interest were involved in PE coding, in each condition.

**Fig. 3:**
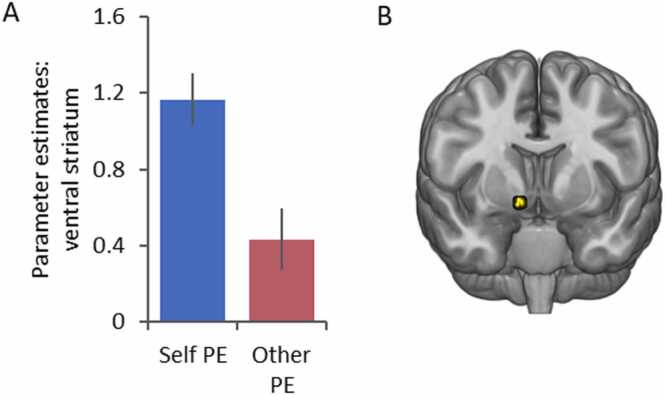
**Ventral striatum response to prediction errors for Self versus Other**. **(A)** Left ventral striatum [x = 12, y = 11, z = − 11] response for Self PE and Other PE. **(B)** Overlay of the response for Self PE > Other PE in the left ventral striatum. All images displayed at *p* < .05 FWE-SVC.

Next, we examined which brain regions responded more to PEs for Self than for Other by contrasting the Self condition against the Other condition (see Supplemental Table S4 for contrasts including the No One condition). The left ventral striatum was the only region to respond more strongly to PEs for Self ([*x* = 12, *y* = 11, *z* = − 11], *Z* = 4.37, *k* = 9, *p* < .001, SVC-FWE; Fig. 3). When examining effects of age we observed no linear or quadratic age-related differences in self-related PE coding. These findings indicate that the ventral striatum responds more to PEs for Self than for Others, and this effect did not differ across age.

**Fig. 4:**
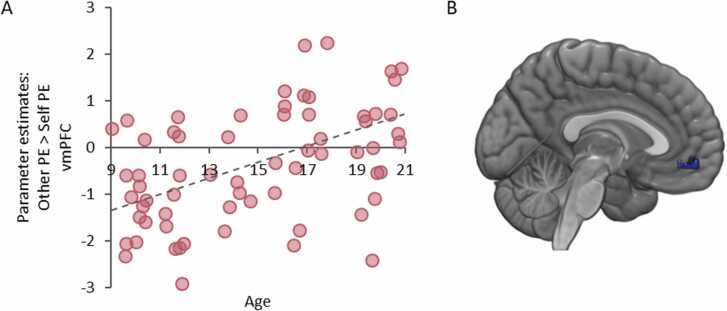
**Linear age effects in responses to Other PE > Self PE in the vmPFC**. **(A)** scatterplot showing the relation between age and activation in the vmPFC for Other PE > Self PE. Scatterplot is only presented for visualization. **(B)** Overlay of the response for Other PE > Self PE in the vmPFC [− 15, 41, − 11]. All images displayed at *p* < .05 FWE-SVC.

We next identified regions that corresponded to PEs for others exclusively by contrasting the Other condition against the Self condition. No voxels in our ROIs responded more strongly to prosocial PEs than Self PEs. When adding age (linear and quadratic) to the model to examine whether age-differences were related to prosocial PE coding, we observed that the vmPFC increasingly responded to prosocial PEs with age ([*x* = − 15, *y* = 50, *z* = 8], *Z* = 4.95, *k* = 45, *p* *=* *.*004, SVC-FWE; see Fig. 4). No effects of quadratic age were observed. This shows that the vmPFC is increasingly involved in prosocial PE coding across adolescence.

### 3.3 Links between cognitive empathy and learning for Others

**Fig. 5:**
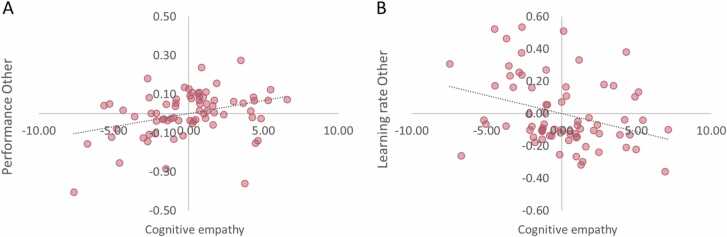
**Relation of cognitive empathy with performance for Others and learning rate for Others**. **(A)** Partial correlation plot showing that individuals with more cognitive empathy perform better for Others (controlled for performance for Self). **(B)** Partial correlation plot showing that individuals with more cognitive empathy have lower learning rates when learning for Others (controlled for learning rate for Self).

Finally, we examined the link between cognitive empathy and prosocial learning. First, we assessed whether cognitive empathy related to performance for Other, while controlling for performance for Self. We observed that individuals with higher empathy ratings, showed better prosocial learning (*r*_*s*_ = 0.30, *p* = .01). Subsequently, we assessed whether cognitive empathy related to learning rate in the Other condition (controlled for learning rate in the Self condition). Results showed that individuals with higher empathy ratings had lower learning rates when learning for Others (cognitive empathy, *r*_*s*_ = − 0.26, *p* = .027, see Fig. 5B). Together, these findings indicate that individuals with more empathy show better learning performance, and integrate information more over trials when learning to benefit others. Finally, we assessed the relation between cognitive empathy and the prosocial PE coding in the vmPFC. For this purpose, we extracted the values of the Other PE > Self PE contrast in vmPFC that showed age-related change (see Fig. 4). Results showed that greater Other vs Self-related PE activation in the vmPFC related to higher empathy scores (cognitive empathy, *r*_*s*_ = 0.31, *p* = .007).

To examine whether age-related differences in empathy or prosocial learning may influence these relations, we additionally included age in the partial correlation analysis. When additionally controlling for age, the relation between empathy and learning for others remained significant (*p* = .029), the relationship between empathy and learning rate became trend-level (*p* = .06), and the relation between empathy and prosocial PE coding was no longer significant (*p* = .15).

## 4 Discussion

This study examined the developmental trajectories of prosocial learning and self-related learning in an adolescent sample spanning ages 9–21 years. We examined the underlying mechanisms in this developmental sample by assessing the neural tracking of PEs during learning for self and others, and how individual differences in cognitive empathy relate to prosocial learning performance. To this end, participants played a two-choice probabilistic reinforcement learning task in which outcomes resulted in monetary consequences for themselves (Self) or an unknown other (Other; prosocial). Our results show improvements in learning for self and others, but the developmental trajectory of prosocial learning is more protracted compared to learning for self. PEs for self were related to activation in the left ventral striatum, which did not show age-related differences. On the other hand, vmPFC-related PE activation during prosocial learning increased with age, and related to individual differences in cognitive empathy. Together, these findings highlight that learning for self and others show different age-related patterns.

The main goal of this study was to examine age-related differences in prosocial learning. Behaviorally, we observed that it is not until mid-adolescence that participants learn similarly for themselves and others. These findings may suggest a self-bias that is stronger in younger ages (van der Aar et al., 2018), and that the motivation to learn for self and others increases with age. Neurally, we observe that a reward-related network including the ventral striatum, sgACC, and vmPFC respond significantly to PEs when learning for Self, Other, and No One. This conjunction presented the starting point for our interest in testing condition-specific learning effects. Contrary to Lockwood et al. (2016), who observed similar PE neural tracking values in the ventral striatum for learning for Self and Others in adults, we observed that PE neural tracking was stronger in the ventral striatum for Self than for Others. Recent reviews, however, suggest that the striatum is related to a range of computations that take place during social learning that could reflect both self-related and other-related learning (Joiner et al., 2017), or the difference between winning for self and others (Báez-Mendoza and Schultz, 2013). Therefore, one explanation for our findings could be related to the possible stronger self-focus or the greater focus on social comparisons reflected in the ventral striatum.

Learning for Others, compared to learning for Self, was associated with stronger activation in the vmPFC with age. Previous prosocial reinforcement learning studies have suggested that the vmPFC is also responsive to processing of self-related expected values (Sul et al., 2015), self-representation (Sui and Humphreys, 2017), or does not differentiate between self and other-related PEs (Lockwood et al., 2016). On the other hand, the vmPFC is suggested to respond to prosocial rewards in adults (Christopoulos and King-Casas, 2015), to others’ outcome PEs (Burke et al., 2010), and to simulated others’ reward PEs (Suzuki et al., 2012). Our findings extend these prior studies by showing that the ventral striatum and vmPFC code PEs both for self and others (see also Joiner et al., 2017). In this first developmental sample investigating prosocial learning, we observe a specificity for Self PEs in the ventral striatum and an increased specificity for prosocial PE coding in the vmPFC, in which across age prosocial (compared to self-related) PE elicit more activation. Alternatively, the pattern of age-related differences we observed for Other and Self-learning in the vmPFC may also support the perspective of a decreasing self-focus with age. For instance, previous work on self-concept development highlights that perspectives of others and self become more merged across development (van der Cruijsen et al., 2019). However, longitudinal studies are more powerful and essential for examining the true developmental trajectories of prosocial learning.

Besides the age-related differences in other-related learning, we observed that consistent with previous findings (Lockwood et al., 2016), individual differences in cognitive empathy were related to prosocial learning. Individuals with higher levels of empathy performed better for Others, integrated outcomes more over time (i.e., lower learning rates), and their vmPFC showed greater activation during prosocial PE coding. However, relations on cognitive empathy and prosocial PE coding in the brain were not robustly observed when controlling for age. This may indicate that it is hard to disentangle whether empathy or age drives prosocial PE coding. Also, age-related differences in brain activity during prosocial PE tracking may be explained by other social cognitive mechanisms than empathy. For instance, although there was no reciprocity or competition, participants may have been influenced by social inequality preferences, such as disliking to getting more (i.e., advantageous inequality aversion), or less (i.e., disadvantageous inequality aversion) than the other participant (Dawes et al., 2007, Fehr and Schmidt, 1999, Meuwese et al., 2015, Westhoff et al., 2020). Future studies could more explicitly assess several social-cognitive skills, strategies, and motivations along with a prosocial learning task to examine what behavioral mechanisms rely most on adolescents’ prosocial learning.

Prior developmental studies on general reinforcement learning remained inconclusive about whether age-related differences were observed in PE neural tracking in the ventral striatum (Christakou et al., 2013, Cohen et al., 2010, Hauser et al., 2015, van den Bos et al., 2012). Here, age-related differences in PE coding for Self were not observed in the ventral striatum. In contrast to other studies (Cohen et al., 2010, Peters and Crone, 2017) we also did not find any quadratic age effects in learning or PE coding. This is possibly due to our narrower age range (9–21 y.o. instead of 8–30 y.o.), as another developmental study on learning also has not observed age-related changes in ventral striatum activity in a similar age range (van den Bos et al., 2012). Indeed, a recent review recommended using samples with wider age ranges, including children and adults, when examining quadratic age effects across adolescence (Li, 2017). It should be noted, however, that although we did not find age-effects in the ventral striatum, the behavioral learning performance for Self showed linear improvements with age. This could also indicate that other mechanisms than simple PE coding may be related to behavioral learning improvement over time within the current age range. For example, a prior study in young adults indicated that besides well-known model-free learning, another more sophisticated and flexible learning system is model-based learning. These two distinct computational strategies use different error signals which are computed in partially distinct brain areas (Gläscher et al., 2010). Moreover, it has been found that people may use different learning strategies, which show different neural activation patterns (Peters et al., 2014b). Future studies are needed to assess whether age-related improvements in learning performance may be more strongly related to strategic learning differences.

We observed that, overall, learning rates decreased with age, and lower learning rates were related to better performance. These findings indicate that, with age, adolescents increasingly integrate information across trials, which was beneficial to their prosocial learning performance. Intriguingly, a recent aging study with a similar prosocial learning task observed that better learning performance was related to *higher* learning rates instead (Cutler et al., 2021, Lockwood et al., 2016). Besides the included age range in this study, a few differences in modeling and task structure may underlie this deviance. First, we allowed a wide range of beta-parameters. Since beta-parameters showed consistent age-related declines (see Supplementary Fig. 2), and also relate to performance (see Supplemental Information) this may have influenced our learning rate estimations. Second, the task structure shows differences in reinforcement structure. Most profoundly we included gains and losses compared to gain and no-gains in previous prosocial learning studies. Possibly, losses may influence the updating of values across trials differently, although we did not find evidence that gains and losses were weighted differently in learning across development. Future studies should further examine the influence of reinforcement structures on observed age-related differences in reinforcement learning.

The current study had several limitations that can be addressed in future research. First, prosocial learning was restricted to unknown others, and participants did not meet these others. Although we circumvented the potential effects of reputational concerns, it may have been more salient to include a confederate, as used in previous studies on prosocial learning in which participants played for a stranger who they met prior to the experimental task (Lockwood et al., 2016, Sul et al., 2015). Second, it would be interesting if future research would extend the prosocial learning task to other beneficiaries. Previous studies have shown that prosocial behaviors and their neural correlates in adolescence strongly depend on the beneficiary (e.g., Brandner et al., 2020, Schreuders et al., 2018, van de Groep et al., 2020, Westhoff et al., 2020). Future studies should further examine whether such differences between beneficiaries are also visible in prosocial *learning* and whether this affects the concurrent neural tracking of PEs. Third, the neural results for the No One condition showed an intermediate pattern between learning for Self and Others, which is difficult to interpret. Behavioral analyses showed that participants generally performed well in this condition (i.e., not significantly different from learning for Self), even though no monetary reinforcers were given depending on task performance. Although including the No One conditions in our contrasts of interest did not alter our main findings, this condition was possibly interpreted by participants in different ways, in which some participants were internally motivated to perform well (e.g., Satterthwaite et al., 2012). Finally, in line with previous research we used a model with separate learning rates per condition (Lockwood et al., 2016). Using this established model, our results also revealed expected differences in learning rate between conditions. However, other studies also included comparison testing whether different learning rates or beta’s are needed across different conditions (Cutler et al., 2021). Future studies may expand on these recent modeling procedure in prosocial learning in developmental and adult populations.

In conclusion, we found that prosocial learning showed age-related improvements across adolescence, suggesting a developmental shift from self-focus in early adolescence to self and other-focus in late adolescence and early adulthood (Crone and Fuligni, 2020). This developmental improvement was associated with stronger recruitment of the vmPFC for others compared to self. This study has implications for learning in social settings, such as educational contexts (Altikulaç et al., 2019), as well as for how children develop prosocial values when learning for unknown others. This study provides the first building blocks to understand age-related differences in how adolescents learn to benefit others.

## CRediT authorship contribution statement

**B.W.**: Conceptualization; Data curation; Formal analysis; Investigation; Project administration; Visualization; Writing – original draft; Writing – review & editing. **N.E.B.**: Investigation; Writing – review & editing. **E.S.**: Investigation; Writing – review & editing. **E.A.C.**: Conceptualization; Funding acquisition; Resources; Writing – original draft; Writing – review & editing. **A.C.K.D.**: Conceptualization; Data curation; Formal analysis; Funding acquisition; Resources; Software; Supervision; Writing - original draft; Writing – review & editing. All authors approved the final version of the manuscript.

## Declaration of Competing Interest

The authors declare that they have no known competing financial interests or personal relationships that could have appeared to influence the work reported in this paper.

## Data Availability

The data that support the findings of this study, and all relevant R codes will be made available in the Leiden repository (https://openaccess.leidenuniv.nl) after acceptance.

## References

[bib1] Altikulaç S., Lee N.C., van der Veen C., Benneker I., Krabbendam L., van Atteveldt N. (2019). The teenage brain: public perceptions of neurocognitive development during adolescence. J. Cogn. Neurosci..

[bib2] Báez-Mendoza R., Schultz W. (2013). The role of the striatum in social behavior. Front. Neurosci..

[bib3] Bates, D., Mächler, M., Bolker, B., & Walker, S., 2014. Fitting linear mixed-effects models using lme4. ArXiv:1406.5823 [Stat]. 〈http://arxiv.org/abs/1406.5823〉.

[bib4] Benjamini Y., Hochberg Y. (1995). Controlling the false discovery rate: a practical and powerful approach to multiple testing. J. R. Stat. Soc. Ser. B (Methodol.).

[bib5] Blakemore S.-J., Mills K.L. (2014). Is adolescence a sensitive period for sociocultural processing?. Annu. Rev. Psychol..

[bib6] Bolenz F., Reiter A.M.F., Eppinger B. (2017). Developmental changes in learning: computational mechanisms and social influences. Front. Psychol..

[bib7] Braams B.R., van Duijvenvoorde A.C.K., Peper J.S., Crone E.A. (2015). Longitudinal changes in adolescent risk-taking: a comprehensive study of neural responses to rewards, pubertal development, and risk-taking behavior. J. Neurosci..

[bib8] Brandner P., Güroğlu B., Crone E.A. (2020). I am happy for us: Neural processing of vicarious joy when winning for parents versus strangers. Cogn. Affect. Behav. Neurosci..

[bib9] Burke C.J., Tobler P.N., Baddeley M., Schultz W. (2010). Neural mechanisms of observational learning. Proc. Natl. Acad. Sci..

[bib10] Casey B.J., Jones R.M., Hare T.A. (2008). The adolescent brain. Ann. N. Y. Acad. Sci..

[bib11] Cheong J.H., Jolly E., Sul S., Chang L.J., Moustafa A.A. (2017). Computational Models of Brain and Behavior.

[bib12] Christakou A., Gershman S.J., Niv Y., Simmons A., Brammer M., Rubia K. (2013). Neural and psychological maturation of decision-making in adolescence and young adulthood. J. Cogn. Neurosci..

[bib13] Christopoulos G.I., King-Casas B. (2015). With you or against you: social orientation dependent learning signals guide actions made for others. NeuroImage.

[bib14] Cohen J.R., Asarnow R.F., Sabb F.W., Bilder R.M., Bookheimer S.Y., Knowlton B.J., Poldrack R.A. (2010). A unique adolescent response to reward prediction errors. Nat. Neurosci..

[bib15] Crone E.A., Dahl R.E. (2012). Understanding adolescence as a period of social–affective engagement and goal flexibility. Nat. Rev. Neurosci..

[bib16] Crone E.A., Fuligni A.J. (2020). Self and others in adolescence. Annu. Rev. Psychol..

[bib17] Cutler J., Wittmann M.K., Abdurahman A., Hargitai L.D., Drew D., Husain M., Lockwood P.L. (2021). Ageing is associated with disrupted reinforcement learning whilst learning to help others is preserved. Nat. Commun..

[bib18] Davidow J.Y., Foerde K., Galván A., Shohamy D. (2016). An upside to reward sensitivity: the hippocampus supports enhanced reinforcement learning in adolescence. Neuron.

[bib19] Davis M.H. (1983). Measuring individual differences in empathy: evidence for a multidimensional approach. J. Personal. Soc. Psychol..

[bib20] Daw N.D., Delgado M.R., Phelps E.A., Robbins T.W. (2011). Decision Making, Affect, and Learning: Attention and Performance XXIII.

[bib21] Dawes C.T., Fowler J.H., Johnson T., McElreath R., Smirnov O. (2007). Egalitarian motives in humans. Nature.

[bib22] den Ouden H.E.M., Daw N.D., Fernandez G., Elshout J.A., Rijpkema M., Hoogman M., Franke B., Cools R. (2013). Dissociable effects of dopamine and serotonin on reversal learning. Neuron.

[bib23] Dumontheil I., Küster O., Apperly I.A., Blakemore S.-J. (2010). Taking perspective into account in a communicative task. NeuroImage.

[bib24] Eisenberg N., Carlo G., Murphy B., Van Court P. (1995). Prosocial development in late adolescence: a longitudinal study. Child Dev..

[bib25] Fehr E., Schmidt K.M. (1999). A theory of fairness, competition, and cooperation. Q. J. Econ..

[bib26] Gläscher J., Daw N., Dayan P., O’Doherty J.P. (2010). States versus rewards: dissociable neural prediction error signals underlying model-based and model-free reinforcement learning. Neuron.

[bib27] Hauser T.U., Iannaccone R., Walitza S., Brandeis D., Brem S. (2015). Cognitive flexibility in adolescence: neural and behavioral mechanisms of reward prediction error processing in adaptive decision making during development. NeuroImage.

[bib28] Hawk S.T., Keijsers L., Branje S.J.T., Graaff J.V., der, Wied M. de, Meeus W. (2013). Examining the Interpersonal Reactivity Index (IRI) among early and late adolescents and their mothers. J. Personal. Assess..

[bib29] Joiner J., Piva M., Turrin C., Chang S.W.C. (2017). Social learning through prediction error in the brain. Npj Sci. Learn..

[bib30] Jones R.M., Somerville L.H., Li J., Ruberry E.J., Powers A., Mehta N., Dyke J., Casey B.J. (2014). Adolescent-specific patterns of behavior and neural activity during social reinforcement learning. Cogn. Affect. Behav. Neurosci..

[bib31] Kim, S., 2015. ppcor: partial and semi-partial (part) correlation (1.1) [Computer software]. 〈https://CRAN.R-project.org/package=ppcor〉.

[bib32] Koller M. (2016). robustlmm: an R package for robust estimation of linear mixed-effects models. J. Stat. Softw..

[bib33] Lenth, R.V., Buerkner, P., Herve, M., Love, J., Riebl, H., Singmann, H., 2021. emmeans: estimated marginal means, aka least-squares means (1.6.3) [Computer software]. 〈https://CRAN.R-project.org/package=emmeans〉.

[bib34] Li R. (2017). Flexing dual-systems models: How variable cognitive control in children informs our understanding of risk-taking across development. Dev. Cogn. Neurosci..

[bib35] Lockwood P.L., Apps M.A.J., Valton V., Viding E., Roiser J.P. (2016). Neurocomputational mechanisms of prosocial learning and links to empathy. Proc. Natl. Acad. Sci..

[bib36] Lockwood P.L., Klein-Flügge M.C. (2020). Computational modelling of social cognition and behaviour—a reinforcement learning primer. Soc. Cogn. Affect. Neurosci..

[bib37] Meuwese R., Crone E.A., de Rooij M., Güroğlu B. (2015). Development of equity preferences in boys and girls across adolescence. Child Dev..

[bib38] Nelson E.E., Leibenluft E., McCLURE E.B., Pine D.S. (2005). The social re-orientation of adolescence: a neuroscience perspective on the process and its relation to psychopathology. Psychol. Med..

[bib39] Netten A.P., Rieffe C., Theunissen S.C.P.M., Soede W., Dirks E., Briaire J.J., Frijns J.H.M. (2015). Low empathy in deaf and hard of hearing (pre)adolescents compared to normal hearing controls. PLoS One.

[bib40] Nussenbaum K., Hartley C.A. (2019). Reinforcement learning across development: what insights can we draw from a decade of research?. Dev. Cogn. Neurosci..

[bib41] Olsson A., Knapska E., Lindström B. (2020). The neural and computational systems of social learning. Nat. Rev. Neurosci..

[bib42] Peters S., Braams B.R., Raijmakers M.E.J., Koolschijn P.C.M.P., Crone E.A. (2014). The neural coding of feedback learning across child and adolescent development. J. Cogn. Neurosci..

[bib43] Peters S., Crone E.A. (2017). Increased striatal activity in adolescence benefits learning. Nat. Commun..

[bib44] Peters S., Koolschijn P.C.M.P., Crone E.A., Van Duijvenvoorde A.C.K., Raijmakers M.E.J. (2014). Strategies influence neural activity for feedback learning across child and adolescent development. Neuropsychologia.

[bib45] Peters S., Van Duijvenvoorde A.C.K., Koolschijn P.C.M.P., Crone E.A. (2016). Longitudinal development of frontoparietal activity during feedback learning: contributions of age, performance, working memory and cortical thickness. Dev. Cogn. Neurosci..

[bib46] Pouw L.B.C., Rieffe C., Oosterveld P., Huskens B., Stockmann L. (2013). Reactive/proactive aggression and affective/cognitive empathy in children with ASD. Res. Dev. Disabil..

[bib47] Pulos S., Elison J., Lennon R. (2004). The hierarchical structure of the Interpersonal Reactivity Index. Soc. Behav. Personal..

[bib48] R Core Team (2020). https://www.R-project.org/.

[bib49] Ruff C.C., Fehr E. (2014). The neurobiology of rewards and values in social decision making. Nat. Rev. Neurosci..

[bib50] Satterthwaite T.D., Ruparel K., Loughead J., Elliott M.A., Gerraty R.T., Calkins M.E., Hakonarson H., Gur R.C., Gur R.E., Wolf D.H. (2012). Being right is its own reward: load and performance related ventral striatum activation to correct responses during a working memory task in youth. NeuroImage.

[bib51] Sawyer S.M., Azzopardi P.S., Wickremarathne D., Patton G.C. (2018). The age of adolescence. Lancet Child Adolesc. Health.

[bib52] Singmann, H., Bolker, B., Westfall, J., Aust, F., & Ben-Sachar, M. S. (2020).*afex: Analysis of Factorial Experiments* (R package version 0.27-2) [R]. https://CRAN.R-project.org/package=afex.https://CRAN.R-project.org/package=afex.

[bib53] Schreuders E., Klapwijk E.T., Will G.-J., Güroğlu B. (2018). Friend versus foe: neural correlates of prosocial decisions for liked and disliked peers. Cogn. Affect. Behav. Neurosci..

[bib54] Steinberg L., Morris A.S. (2001). Adolescent development. Annu. Rev. Psychol..

[bib55] Sui J., Humphreys G.W. (2017). The ubiquitous self: what the properties of self-bias tell us about the self. Ann. N. Y. Acad. Sci..

[bib56] Sul S., Tobler P.N., Hein G., Leiberg S., Jung D., Fehr E., Kim H. (2015). Spatial gradient in value representation along the medial prefrontal cortex reflects individual differences in prosociality. Proc. Natl. Acad. Sci..

[bib57] Suzuki S., Harasawa N., Ueno K., Gardner J.L., Ichinohe N., Haruno M., Cheng K., Nakahara H. (2012). Learning to simulate others’ decisions. Neuron.

[bib58] van de Groep S., Meuwese R., Zanolie K., Güroğlu B., Crone E.A. (2018). Developmental changes and individual differences in trust and reciprocity in adolescence. J. Res. Adolesc..

[bib59] van de Groep S., Zanolie K., Crone E.A. (2020). Giving to friends, classmates, and strangers in adolescence. J. Res. Adolesc..

[bib60] van den Bos W. (2009). Better than expected or as bad as you thought? The neurocognitive development of probabilistic feedback processing. Front. Hum. Neurosci..

[bib61] van den Bos W., Cohen M.X., Kahnt T., Crone E.A. (2012). Striatum–medial prefrontal cortex connectivity predicts developmental changes in reinforcement learning. Cereb. Cortex.

[bib62] van der Aar L.P.E., Peters S., Crone E.A. (2018). The development of self-views across adolescence: Investigating self-descriptions with and without social comparison using a novel experimental paradigm. Cogn. Dev..

[bib63] van der Cruijsen R., Peters S., Zoetendaal K.P.M., Pfeifer J.H., Crone E.A. (2019). Direct and reflected self-concept show increasing similarity across adolescence: a functional neuroimaging study—ScienceDirect. Neuropsychologia.

[bib64] van Duijvenvoorde A.C.K., Achterberg M., Braams B.R., Peters S., Crone E.A. (2016). Testing a dual-systems model of adolescent brain development using resting-state connectivity analyses. NeuroImage.

[bib65] van Duijvenvoorde A.C.K., Op de Macks Z.A., Overgaauw S., Gunther Moor B., Dahl R.E., Crone E.A. (2014). A cross-sectional and longitudinal analysis of reward-related brain activation: effects of age, pubertal stage, and reward sensitivity. Brain Cogn..

[bib66] van Duijvenvoorde A.C.K., Westhoff B., Vos F., Wierenga L.M., Crone E.A. (2019). A three‐wave longitudinal study of subcortical–cortical resting‐state connectivity in adolescence: testing age‐ and puberty‐related changes. Hum. Brain Mapp..

[bib67] Westhoff B., Molleman L., Viding E., van den Bos W., van Duijvenvoorde A.C.K. (2020). Developmental asymmetries in learning to adjust to cooperative and uncooperative environments. Sci. Rep..

