# Supplementary material for: Increased Ventromedial Prefrontal Cortex Activity in Adolescence Benefits Prosocial Reinforcement Learning
Source: Dev Cogn Neurosci. 2021 Oct 2;52:101018. doi: 10.1016/j.dcn.2021.101018 (PMC8529395; doi:10.1016/j.dcn.2021.101018)
Supplement: Supplementary file 1 — Supplementary material [file mmc1.docx]

# **Supplementary information for**

**Increased ventromedial prefrontal cortex activity in adolescence**

**benefits prosocial reinforcement learning**

Bianca Westhoff^1,2*^; Neeltje E. Blankenstein^3^; Elisabeth Schreuders^1,2,4^; Eveline A. Crone^1,5^; Anna C. K. van Duijvenvoorde^1,2*^

^1^ Institute of Psychology, Leiden University, Wassenaarseweg 52, 2333 AK Leiden, Netherlands

^2^ Leiden Institute for Brain and Cognition, Leiden, Netherlands

^3^ Amsterdam UMC, Vrije Universiteit Amsterdam, Child and Adolescent Psychiatry, de Boelelaan 1117, Amsterdam, Netherlands

^4^ Department of Clinical, Neuro and Developmental Psychology, Vrije Universiteit, Amsterdam, Netherlands

^5^ Erasmus School of Social and Behavioural Sciences, Erasmus University Rotterdam, Netherlands

*** Correspondence should be addressed to:**

Bianca Westhoff (b.westhoff@fsw.leidenuniv.nl)

Anna van Duijvenvoorde (a.c.k.van.duijvenvoorde@fsw.leidenuniv.nl)

Institute of Psychology, Leiden University, Wassenaarseweg 52, 2333 AK Leiden, Netherlands

#### Figure S1. Number of participants across age per sex. In total, 74 participants were included (39 female, 35 male).

### Beta parameter

The Beta parameter was examined as an index to what extent participants followed expected value in their choice behavior, and is also considered a parameter of decision noise. Higher values represent less decision noise here. Using a robust linear mixed effects model, we assessed effects of Condition and Age (linear) in beta parameters. We observed that with increasing age, decision noise decreased linearly (main effect of Age, *B = 2.9, p* = 0.007), indicating participants follow expected value more closely. Particularly beta parameters increased more strongly across age for Other than for Self (Other-Self; *B* = 0.47, *p* < .001), and did not differ significantly between No One and Other (NoOne – Other *p* = .19) and between No One and Self (No One – Self; *p* = .19)

■■■ 9-11 y.o.

■■■ 13-17 y.o.

■■■ 19-21 y.o.

#### Figure S2. Beta parameter per condition per age cohort.

Age is used as a continuous variable in all analyses, but is visualized as age cohorts for illustrative purposes and interpretability.

■■■ 9-11 y.o.

■■■ 13-17 y.o.

■■■ 19-21 y.o.

#### Figure S3. BIC values per condition per age cohort.

Bars show BIC differences of the two-learning rate model (gain and loss) with the best model (one learning rate). Values on the y-axis indicate the difference between fit values (BIC values) for the two-learning rate model (gains and loss) and fit values for the one-learning rate model (the best model). BIC values were calculated per participant, and are shown separately per age cohort and per condition. For all age cohorts and all conditions a one-learning rate model is the best-fitting model. Lower bars indicate that the model fit of the one-learning rate model and two-learning rate model are more similar. For each condition, these BIC difference scores are not predicted by age (all *Ps* > .14). Error bars represent standard error of the mean.

### Relations between performance, learning rates and betas

We tested non-parametric correlations between performance, learning rates, and betas per condition. These show that lower learning rates in the Other condition are related to better performance for Other (rs(74) = -.38, *p* = .001). Similarly, lower learning rates in the No One condition are related to better learning for No One (rs(74) = -.34, *p* = .003, but learning rates in the Self condition are not significantly related to learning for Self (rs(74) = -.22, *p* = .056). In addition, higher betas were strongly related to better performance in all conditions (Self, rs (74) = .91, *p* < .001; Other rsn(74) = .94, *p* <.001; No One rs (74) = .89, *p* < .001. Also, in all conditions, lower learning rates are related to higher betas (Self, rs(74) = -.37, p < .001; Other, rs(74) = -.54, p < .001; No One, rs(74) = -.48, p < .001).

**
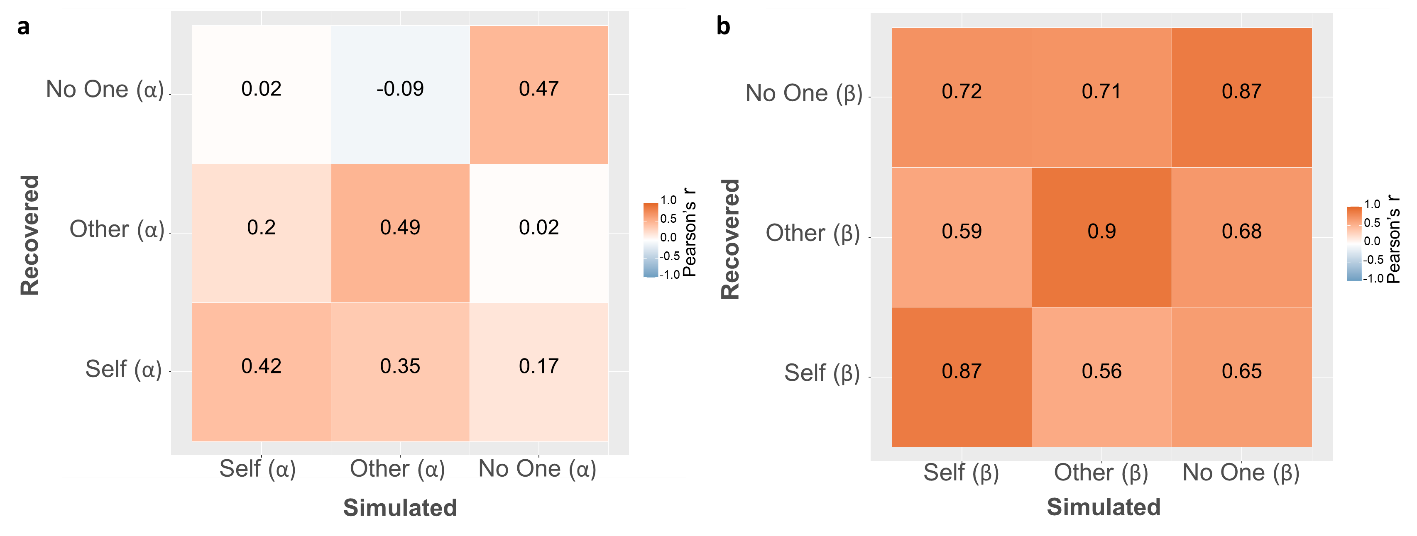
**

#### Figure S4. Learning rate and Beta parameter recovery. The correlation matrices represent the correlations between simulated and recovered (a) learning rates, and (b) beta values. Stronger colors show higher values and high values on the diagonal show parameters can be recovered.

####
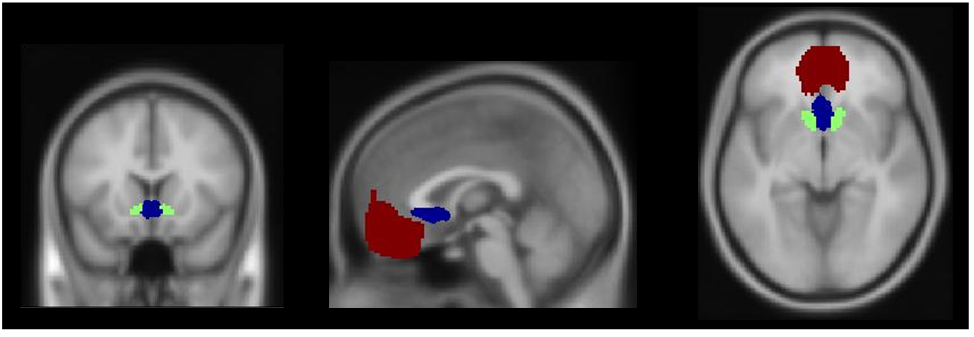
Figure S5. Regions of interest.

Ventromedial prefrontal cortex (vmPFC; red), subgenual anterior cingulate cortex (sgACC; blue), and the ventral striatum (green).

| **A Self PE**  **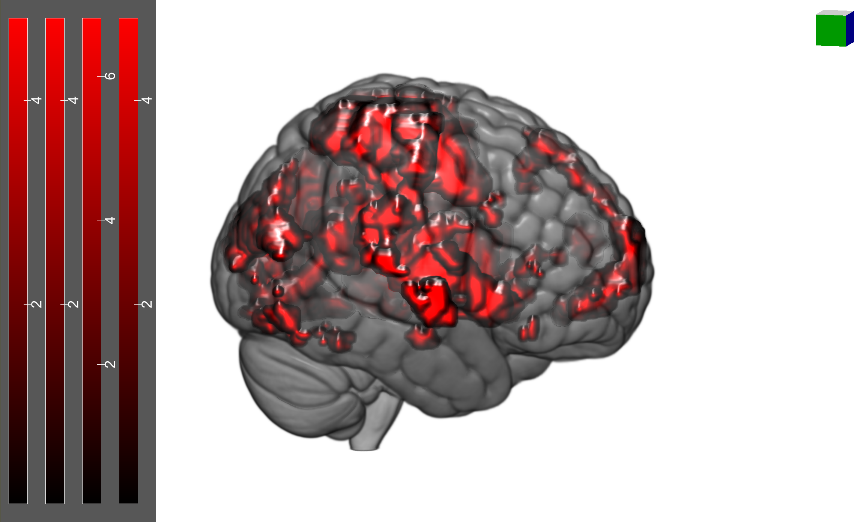 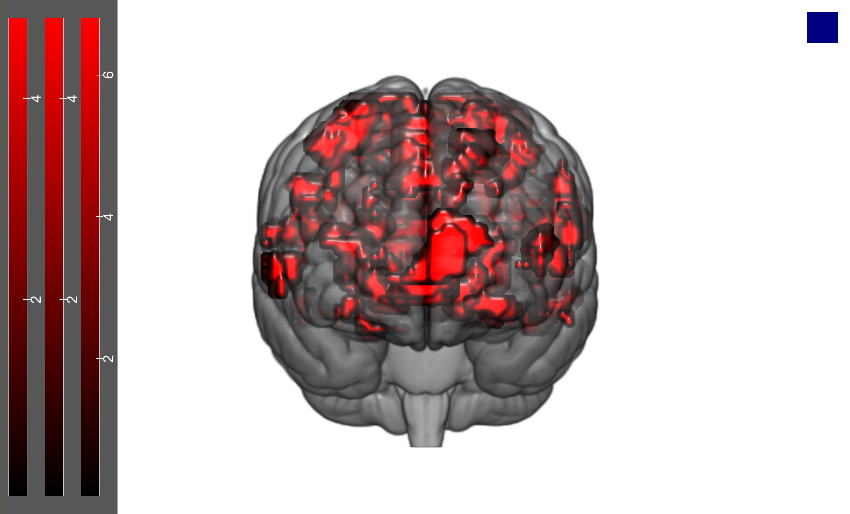 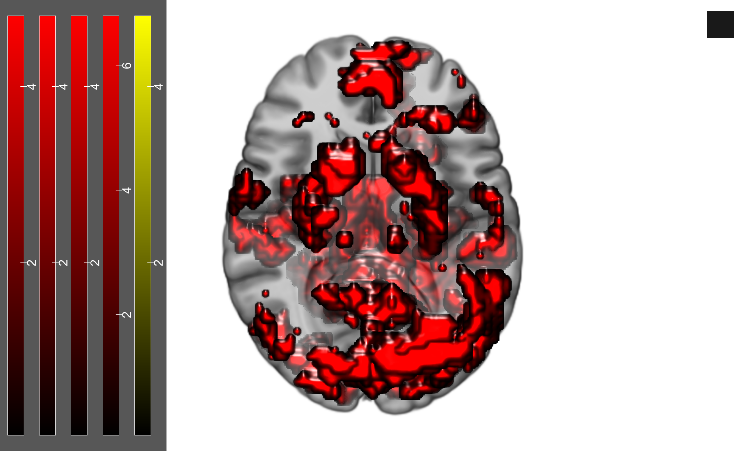**  **B Other PE**  **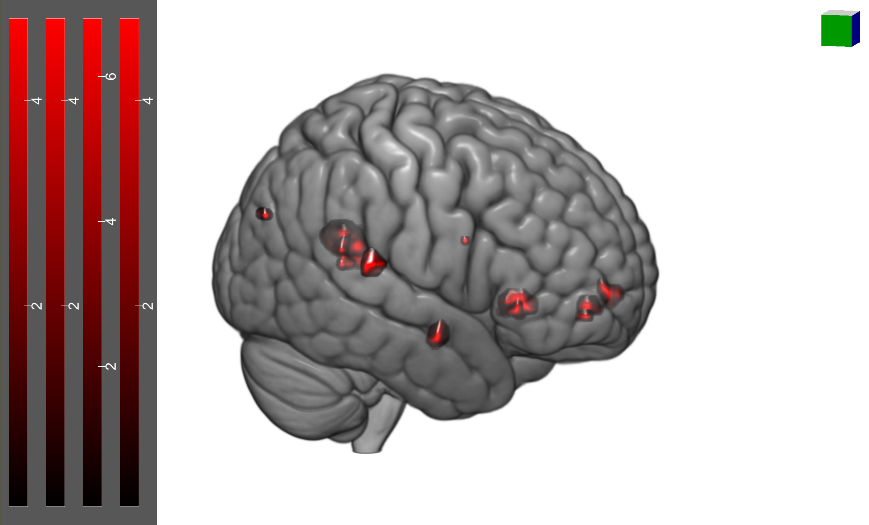 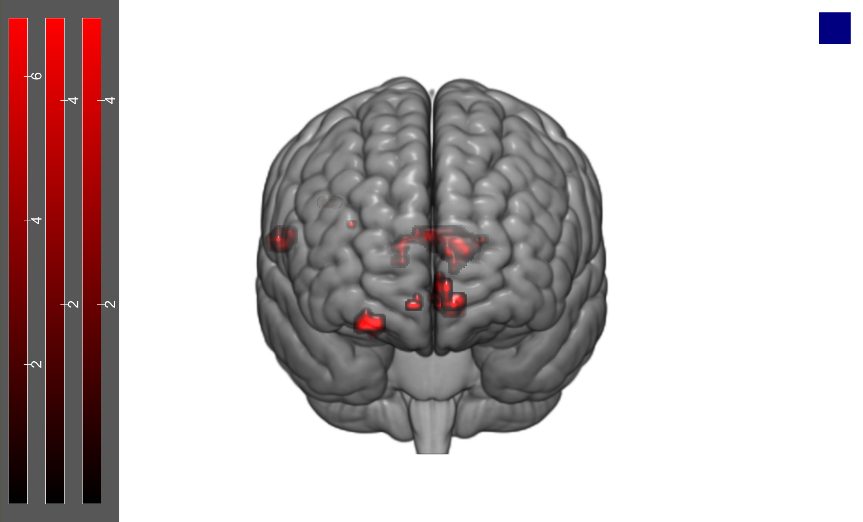 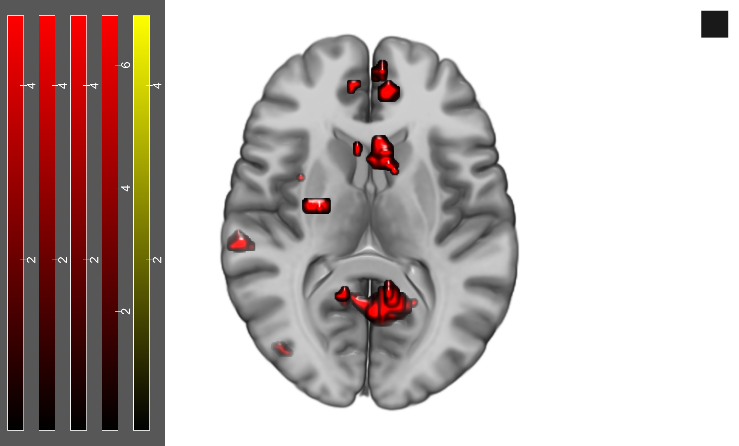**  **C No One PE**  **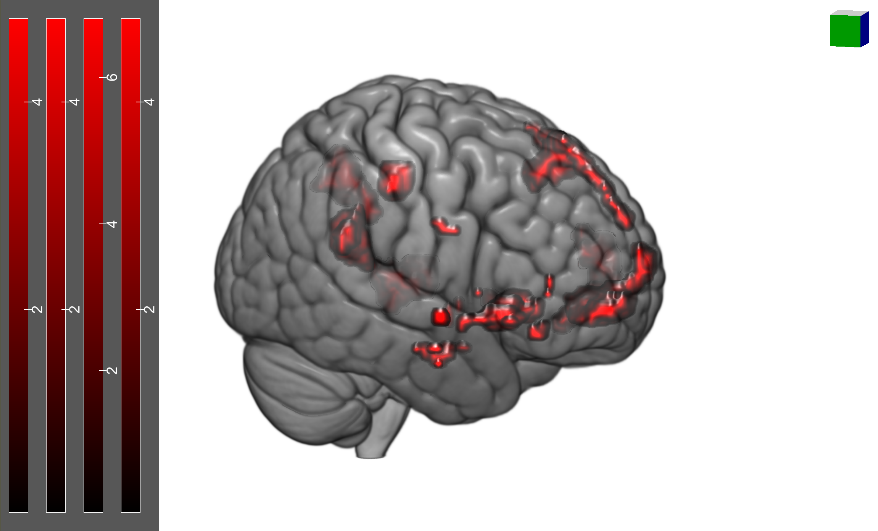 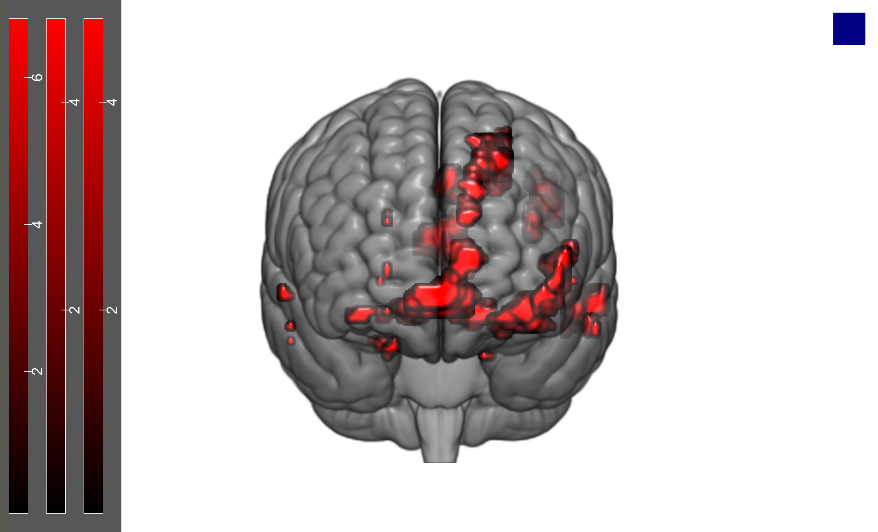 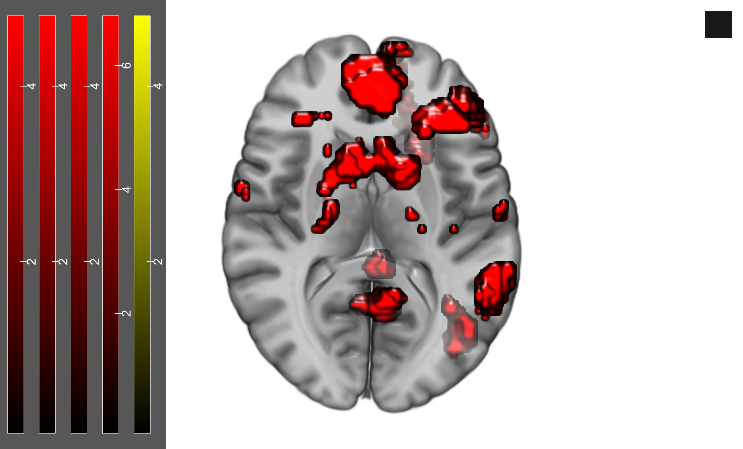**  **D Conjunction PE**  **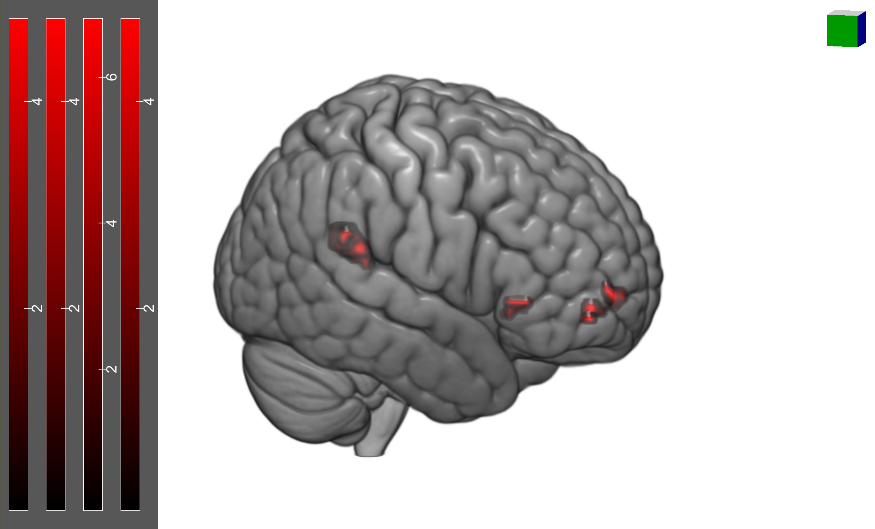 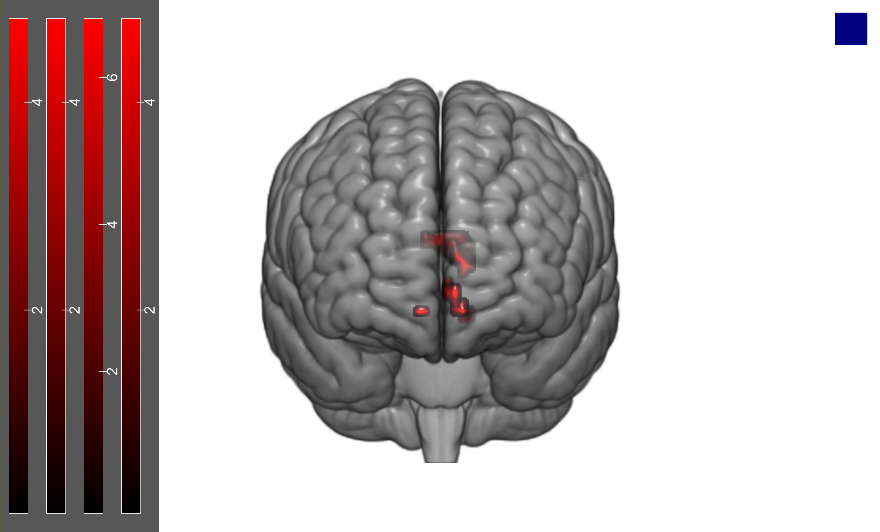 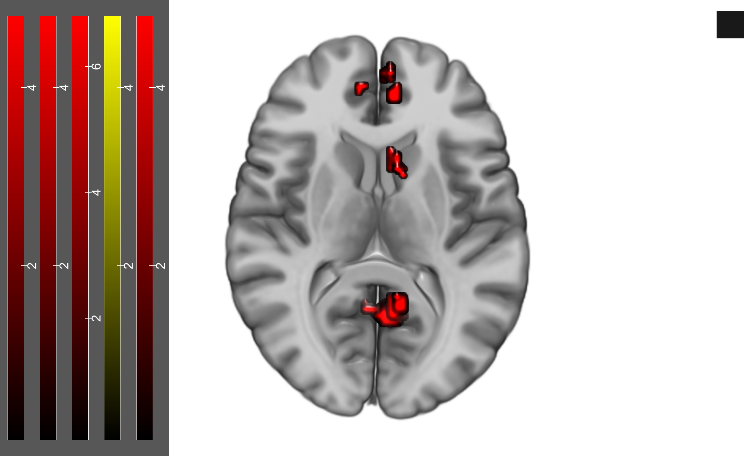** |
| --- |

#### **Figure S6.** Whole brain responses for (A) Self PE, (B) Other PE, (C) No One PE, and (D) conjunction (common PE coding in all three conditions). All images displayed at p < .05 FWE, voxel level corrected.

**
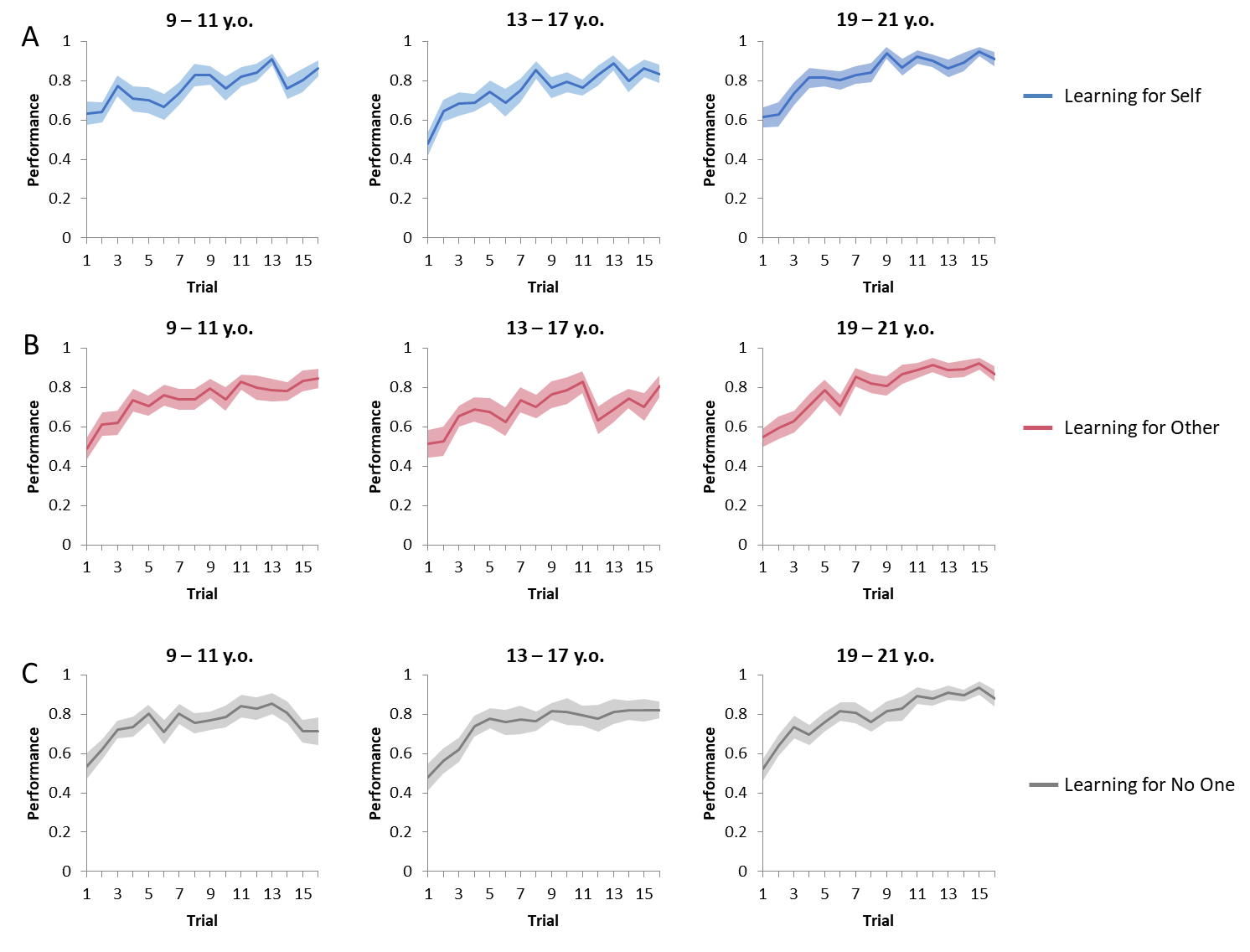
**

#### Figure S7. Learning across trials for (A) Self, (B) Others, and (C) No One, per age cohort.

Note that for all analyses including age, we used age as a continuous variable. However, figures represent age per age cohorts instead for illustrative purposes and interpretability.

#### Figure S8. Cognitive empathy across age.

#### Table S1. Mean model parameters with prior distributions (M, SD), constraint, and 95% confidence intervals around the mean.

| Model | Parameter | Prior | Constraint | Mean | 95% Confidence Interval for Mean |
| --- | --- | --- | --- | --- | --- |
|  |  |  |  |  |  |
| RL Self | α | β (1.2,1.2) | 0 < α < 1 | 0.28 | .24 – .32 |
|  | β | Gaussian (0,10) | −∞ ≤ β ≤ ∞ | 8.81 | 7.37 – 10.26 |
|  |  |  |  |  |  |
|  |  |  |  |  |  |
| RL Other | α | β (1.2,1.2) | 0 < α < 1 | 0.34 | .29 – .39 |
|  | β | Gaussian (0,10) | −∞ ≤ β ≤ ∞ | 7.43 | 5.92 – 8.94 |
|  |  |  |  |  |  |
|  |  |  |  |  |  |
| RL NoOne | α | β (1.2,1.2) | 0 < α < 1 | 0.34 | .29 – .39 |
|  | β | Gaussian (0,10) | −∞ ≤ β ≤ ∞ | 7.94 | 6.52 – 9.37 |

#### Table S2. Main effects of whole brain prediction error responses per condition, and common prediction error coding (conjunction).

|  | | *Peak voxel* | | |  |  |  |
| --- | --- | --- | --- | --- | --- | --- | --- |
| *Brain Region* | | *x* | *y* | *z* | *k* | *t* | *z* |
| **Main effect Self PE** | | | | | | | |
|  | L Precental gyrus | -27 | -25 | 61 | 6235 | 9.68 | Inf |
|  | L precuneus | -3 | -58 | 13 |  | 9.65 | Inf |
|  | L Postcentral gyrus | -30 | -34 | 64 |  | 9.03 | Inf |
|  | L Putamen | -30 | -13 | 4 | 481 | 9.52 | Inf |
|  | L Putamen | -15 | 8 | -11 |  | 8.20 | 7.65 |
|  | R Caudate | 12 | 11 | -11 | 525 | 9.01 | Inf |
|  | R Thalamus | 30 | -16 | 7 |  | 8.22 | 7.65 |
|  | R Putamen | 27 | -7 | 10 |  | 8.04 | 7.52 |
|  | L Superior frontal gyrus, medial | -9 | 65 | 19 | 515 | 7.25 | 6.85 |
|  | L Superior frontal gyrus, medial | -12 | 58 | 7 |  | 6.93 | 6.58 |
|  | L Superior frontal gyrus, medial | 0 | 59 | 1 |  | 6.85 | 6.52 |
|  | L Rolandic operculum | -48 | -28 | 19 | 271 | 6.95 | 6.60 |
|  | L Superior temporal gyrus | -60 | -31 | 19 |  | 6.71 | 6.39 |
|  | L Supramarginal gyrus | -60 | -22 | 16 |  | 6.57 | 6.27 |
|  | L Middle frontal gyrus, orbital part | -30 | 35 | -14 | 50 | 6.73 | 6.41 |
|  | L Thalamus | -12 | -22 | 4 | 21 | 6.57 | 6.27 |
|  | R Superior temporal gyrus | 63 | -1 | -5 | 83 | 6.52 | 6.23 |
|  | R Rolandic operculum | 63 | 5 | 1 |  | 6.06 | 5.82 |
|  | R Superior temporal gyrus | 66 | -7 | 4 |  | 5.29 | 5.13 |
|  | L Inferior frontal gyrus,triangular part | -48 | 32 | 13 | 60 | 6.47 | 6.19 |
|  | L Rolandic operculum | -54 | -4 | 4 | 34 | 6.04 | 5.81 |
|  | R Inferior temporal gyrus | 51 | -67 | -11 | 46 | 5.76 | 5.55 |
|  | R Inferior temporal gyrus | 51 | -58 | -20 |  | 5.41 | 5.23 |
|  | R Inferior occipital gyrus | 42 | -76 | -17 |  | 5.35 | 5.19 |
|  | R Cerebellum | 21 | -52 | -23 | 20 | 5.60 | 5.41 |
| **Main effect Other PE** | | | | | | | |
|  | L Precuneus | -6 | -61 | 13 | 103 | 6.54 | 6.25 |
|  | L Calcarine fissure & surrounding cortex | -12 | -55 | 10 |  | 6.19 | 5.94 |
|  | L Olfactory cortex | -6 | 20 | -11 | 32 | 6.29 | 6.02 |
|  | R Hippocampus | 30 | -7 | -20 | 17 | 5.66 | 5.46 |
|  | R Superior temporal gyrus | 63 | -28 | 16 | 17 | 5.59 | 5.40 |
|  | L Middle frontal gyrus, orbital part | -9 | 44 | -11 | 12 | 5.58 | 5.39 |
| **Main effect No One PE** | | | | | | | |
|  | L Middle frontal gyrus, orbital part | -3 | 50 | -11 | 246 | 9.13 | Inf |
|  | L Superior frontal gyrus, medial | -6 | 62 | 1 |  | 5.92 | 5.69 |
|  | L Superior frontal gyrus, medial | -9 | 55 | 13 |  | 5.77 | 5.56 |
|  | R Caudate | 12 | 8 | -11 | 173 | 7.64 | 7.19 |
|  | L Olfactory cortex | -15 | 11 | -14 |  | 6.72 | 6.40 |
|  | L Olfactory cortex | -5 | 20 | -11 |  | 6.19 | 5.93 |
|  | L Inferior frontal gyrus, orbital part | -36 | 35 | -14 | 182 | 7.60 | 7.15 |
|  | L Middle frontal gyrus, orbital part | -24 | 32 | -17 |  | 6.30 | 6.04 |
|  | L Inferior frontal gyrus, triangular part | -45 | 32 | 7 |  | 5.99 | 5.75 |
|  | L Middle temporal gyrus | -60 | -43 | -8 | 134 | 7.21 | 6.82 |
|  | L Inferior temporal gyrus | -54 | -52 | -17 |  | 6.39 | 6.09 |
|  | L Precuneus | -6 | -55 | 16 | 120 | 7.16 | 6.78 |
|  | L Calcarine fissure & surrounding cortex | -12 | -52 | 7 |  | 5.88 | 5.66 |
|  | L Median cingulate and paracingulate gyri | -3 | -37 | 40 | 50 | 6.57 | 6.27 |
|  | L Middle frontal gyrus | -24 | 32 | 49 | 172 | 6.56 | 6.26 |
|  | L Middle frontal gyrus | -24 | 20 | 46 |  | 5.99 | 5.75 |
|  | Superior frontal gyrus, medial | -12 | 59 | 28 |  | 5.64 | 5.45 |
|  | L Angular gyrus | -42 | -67 | 34 | 122 | 6.14 | 5.89 |
|  | R Parahippocampal gyrus | 18 | -10 | -26 | 16 | 5.63 | 5.43 |
|  | R Parahippocampal gyrus | 24 | -19 | -23 |  | 5.20 | 5.04 |
| **Conjunction** | | | | | | | |
|  | L Precuneus | -6 | -58 | 16 | 61 | 6.54 | 6.24 |
|  | L Caudate | -6 | 14 | -8 | 12 | 5.67 | 5.49 |

For all regions, FWE *p* < .05 voxel-level whole-brain corrected, and presented here with *k* > 10. PE = Prediction error; L = Left; R = Right; k =cluster extent. Names of the brain regions derived from the Automated Anatomical Labeling (AAL) atlas.

#### Table S3. Main effects of whole brain expected value responses per condition, and common expected value coding (conjunction).

|  | | *Peak voxel* | | |  |  |  |
| --- | --- | --- | --- | --- | --- | --- | --- |
| *Brain Region* | | *x* | *y* | *z* | *k* | *t* | *z* |
| **Main effect Self EV** | | | | | | | |
|  | Precuneus | -15 | -64 | 19 | 14 | 5.63 | 5.44 |
| **Main effect Other EV** | | | | | | | |
|  | L Precuneus | -9 | -61 | 19 | 53 | 6.01 | 5.77 |
|  | R Middle frontal gyrus, orbital part | 3 | 56 | -5 | 23 | 5.51 | 5.32 |
| **Main effect No One EV** | | | | | | | |
|  | L Middle temporal gyrus | -51 | -13 | -8 | 11 | 5.36 | 5.19 |
| **Conjunction*** | | | | | | | |
|  | L Precuneus | -12 | -58 | 13 | 140 | 4.28 | 4.18 |
|  | L Precuneus | -12 | -58 | 22 |  | 3.99 | 3.91 |
|  | R Precuneus | 6 | -58 | 19 |  | 4.13 | 4.04 |

For all regions, FWE *p* < .05 voxel-level whole-brain corrected, and presented here with *k* > 10. PE = Prediction error; L = Left; R = Right; k =cluster extent. Names of the brain regions derived from the Automated Anatomical Labeling (AAL) atlas. *threshold *p* < .001

#### Table S4. Comparison of responses to prediction errors between conditions, in regions of interest (ventral striatum, sgACC, vmPFC).

|  | | *Peak voxel* | | |  |  |  |
| --- | --- | --- | --- | --- | --- | --- | --- |
| *Brain Region* | | *x* | *y* | *z* | *k* | t | z |
| **No One PE > Other PE** | |  |  |  |  |  |  |
|  | Ventral striatum | 12 | 8 | -11 | 2 | 3.41 | 3.36 |
| **No One PE > Self PE** | |  |  |  |  |  |  |
|  | No suprathreshold voxels |  |  |  |  |  |  |
| **Other PE > Self PE + No One PE** | |  |  |  |  |  |  |
|  | No suprathreshold voxels |  |  |  |  |  |  |
| **Self PE + No One PE > Other PE** | |  |  |  |  |  |  |
|  | Ventral striatum | 12 | 8 | -11 | 8 | 4.52 | 4.42 |
|  | sgACC | 9 | 8 | -11 | 2 | 3.75 | 3.69 |
| **Self PE > Other PE + No One PE** | |  |  |  |  |  |  |
|  | Ventral striatum | 12 | 11 | -11 | 3 | 3.34 | 3.30 |
| **No One PE > Self PE + Other PE** | |  |  |  |  |  |  |
|  | No suprathreshold voxels |  |  |  |  |  |  |
| **Self PE + Other PE > No One PE** | |  |  |  |  |  |  |
|  | No suprathreshold voxels |  |  |  |  |  |  |
| **Other PE + No One PE > Self PE** | |  |  |  |  |  |  |
|  | No suprathreshold voxels |  |  |  |  |  |  |

For all regions, corrected at *p* < .05 FWE-SVC. PE = Prediction error; k =cluster extent. Names of the brain regions were based on the Automated Anatomical Labeling (AAL) atlas.

#### Table S5. Comparison of whole brain responses to prediction errors between conditions

|  | | *Peak voxel* | | |  |  |  |
| --- | --- | --- | --- | --- | --- | --- | --- |
| *Brain Region* | | *x* | *y* | *z* | *k* | t | z |
| **No One PE > Other PE** | |  |  |  |  |  |  |
|  | No suprathreshold voxels |  |  |  |  |  |  |
| **No One PE > Self PE** | |  |  |  |  |  |  |
|  | No suprathreshold voxels |  |  |  |  |  |  |
| **Other PE > Self PE + No One PE** | |  |  |  |  |  |  |
|  | No suprathreshold voxels |  |  |  |  |  |  |
| **Self PE + No One PE > Other PE** | |  |  |  |  |  |  |
|  | R Precuneus | 15 | -100 | 13 | 27 | 4.51 | 4.23 |
|  | R Calcarine | 18 | -100 | 1 |  | 3.7 | 3.53 |
|  | L Calcarine | -12 | -103 | -5 | 20 | 3.90 | 3.70 |
|  | L Calcarine | -6 | -103 | 1 |  | 3.73 | 3.55 |
|  | L Postcentral | -36 | -34 | 67 | 15 | 3.84 | 3.65 |
|  | L Caudate | -12 | -7 | 22 | 13 | 3.81 | 3.63 |
|  | L Thalamus | -12 | -16 | 13 |  | 3.68 | 3.51 |
| **Self PE > Other PE + No One PE** | |  |  |  |  |  |  |
|  | L Occipital gyrus | -21 | -88 | 22 | 2385 | 6.17 | 5.52 |
|  | L Cerebellum | -33 | -64 | -20 |  | 5.02 | 4.64 |
|  | R Occipital gyrus | 24 | -88 | 19 |  | 5.00 | 4.62 |
|  | L Postcentral | -30 | -37 | 67 | 701 | 5.31 | 4.87 |
|  | L Precentral | -21 | -25 | 58 |  | 4.85 | 4.50 |
|  | R Precuneus | 6 | -43 | 58 |  | 4.63 | 4.32 |
|  | R Putamen | 33 | -13 | 4 | 261 | 4.57 | 4.27 |
|  | R putamen | 30 | -22 | 4 |  | 4.57 | 4.27 |
|  | L Putamen | -30 | -13 | 4 | 366 | 4.50 | 4.21 |
|  | L Supramarginal gyrus | -57 | -28 | 28 |  | 4.15 | 3.92 |
|  | R Thalamus | 3 | -13 | 55 | 165 | 4.49 | 4.21 |
|  | L Supplementary motor area | -3 | -19 | 58 |  | 4.42 | 4.14 |
|  | Median cingulate and paracingulate gyri | 6 | -1 | 43 |  | 4.13 | 3.90 |
|  | R Middle frontal gyrus | 36 | 50 | 31 | 22 | 4.13 | 3.90 |
|  | R Supramarginal gyrus | 45 | -28 | 34 | 107 | 4.30 | 4.05 |
|  | R Supramarginal gyrus | 54 | -28 | 37 |  | 4.02 | 3.81 |
|  | R Superior frontal gyrus, dorsolateral | 27 | -10 | 70 | 33 | 4.25 | 4.00 |
|  | L Superior temporal gyrus | -54 | -4 | 4 | 24 | 4.04 | 3.83 |
|  | L Putamen | -15 | 11 | -11 | 11 | 4.02 | 3.81 |
|  | R Precental gyrus | 42 | -10 | 58 | 25 | 3.89 | 3.69 |
|  | R Precental gyrus | 39 | -10 | 49 |  | 3.83 | 3.64 |
|  | R Thalamus | 15 | -22 | 1 | 10 | 3.75 | 3.57 |
| **No One PE > Self PE + Other PE** | |  |  |  |  |  |  |
|  | No suprathreshold voxels |  |  |  |  |  |  |
| **Self PE + Other PE > No One PE** | |  |  |  |  |  |  |
|  | R Precuneus | 21 | -64 | 25 | 99 | 4.63 | 4.32 |
|  | R Calcarine fissure and surrounding cortex | 21 | -61 | 16 |  | 4.59 | 4.29 |
|  | R Cuneus | 15 | -82 | 28 |  | 3.72 | 3.54 |
|  | L Cuneus | -9 | -82 | 22 | 75 | 4.33 | 4.07 |
|  | L Cuneus | -15 | -67 | 19 |  | 4.12 | 3.90 |
|  | L Cuneus | -15 | -82 | 31 |  | 3.29 | 3.17 |
|  | R Insula | 36 | 8 | 4 | 58 | 3.80 | 3.62 |
|  | R Insula | 36 | 2 | 16 |  | 3.61 | 3.46 |
| R Supramarginal gyrus | | 66 | -25 | 22 | 61 | 4.05 | 3.84 |
| R Superior temporal gyrus | | 45 | -34 | 22 |  | 3.58 | 3.57 |
| R Supramarginal gyrus | | 54 | -34 | 31 |  | 3.57 | 3.42 |
| R Heschl gyrus | | 45 | -25 | 13 | 18 | 3.92 | 3.72 |
| L Middle occipital gyrus | | -30 | -76 | 22 | 12 | 3.67 | 3.50 |
| L Middle temporal gyrus | | -57 | -67 | 4 | 10 | 3.62 | 3.46 |
| **Other PE + No One PE > Self PE** | |  |  |  |  |  |  |
|  | No suprathreshold voxels |  |  |  |  |  |  |

For all regions, *p* < .001 voxel-level uncorrected, extent-threshold *k* = 10. Names of the brain regions were based on the Automated Anatomical Labeling (AAL) atlas.

### Participant instructions

“Welcome! We are going to play a game in the scanner. In this game, you will see two pictures on the screen. You can win or lose points by choosing one of the pictures. If you win, you get +1 point, and if you lose, you get -1 point. But not all pictures are equally good…”

“With both pictures you can win and lose, but with one picture you will win more often, and with the other picture you will lose more often. Try to win as many points as possible! Note: it does not matter whether the picture is on the left or right side of the screen.”

“To choose the left picture, you press the left button. To choose the right picture, you press the right button. At the end of the game, you will see how many points you won in total. Your points will be translated to real money using a formula. This amount of money will be paid out to you.”

“You will play this game 3 times: for yourself, for another person, and for no one. On the screen, it says for whom you will be playing. Each time you should learn which of the two pictures on the screen is better. Sometimes you play for yourself. When you play for yourself, the gains will be paid out to you.”

“Sometimes you play for another person. When you play for another person, the gains will be paid out to another player. This player is someone who participates in this experiment after you. This is a girl or a boy of your age. This person does not know that you are playing for him/her. So, he/she will receive the money you win for him/her without them knowing it is from you. This person will not play the game for you.”

“Sometimes you play for no one. When you play for no one, your points don’t count and no one will receive your gains.”

“Try to respond on time. You will have about 2 seconds to make your choice. We will first do a practice run. Good luck!”

*[24 Practice trials (8 per condition)]*

“Well done! This was a practice run, so your points don’t count yet. In the scanner, we will play the game for real, and you will see at the end of the game how many points you won for yourself and for the other person. Do you have any questions left?”
